# User Testing to Improve Retrieval and Comprehension of Information in Guidelines to Improve Medicines Safety

**DOI:** 10.1097/PTS.0000000000000723

**Published:** 2020-06-22

**Authors:** Matthew D. Jones, Bryony Dean Franklin, Margaret C. Watson, DK Raynor

**Affiliations:** From the ∗ Department of Pharmacy and Pharmacology, University of Bath, Bath; † UCL School of Pharmacy, London; ‡ Strathclyde Institute of Pharmacy and Biomedical Sciences, University of Strathclyde, Glasgow; § Luto Research; ∥ School of Healthcare, University of Leeds, Leeds, United Kingdom.

**Keywords:** guidelines, medicines safety, medication errors, user testing, information design, intravenous, injectable medicines, nurses, usability, user experience

## Abstract

**Objective:**

The aim of the study was to investigate the effectiveness of user testing for improving healthcare professionals’ retrieval and comprehension of information in medicines guidelines.

**Methods:**

The United Kingdom’s Injectable Medicines Guide was selected as a case study. This gives guidance to nurses on preparing and administering intravenous medicines on hospital wards, in line with standard UK practice. Three rounds of user testing were completed with 10 hospital nurses per round, using the Injectable Medicines Guide for voriconazole and aminophylline. Participants used the guidelines to answer 17 questions related to the administration of these medicines. Answers were scored for “finding” and “understanding” the required information. Semistructured interviews explored participants’ opinions of guideline content, design, and wording, with responses analyzed thematically. The guidelines were revised between rounds.

**Results:**

In round 1, 8 of 17 questions were answered correctly by all participants. Participants had difficulty with dose, dilution, administration rate, and adverse effects questions. Revisions included new subsections and increased calculation support. In round 2, 14 of 17 questions were answered correctly by all participants. Difficulty persisted with dose and administration rate questions and further revisions made. In round 3, 15 of 17 questions were answered correctly by all participants. Across all rounds, participants considered appropriate subheadings and information order as important for fast location of information. Specific, detailed, and practical instructions were perceived as important to improve understandability and usefulness.

**Conclusions:**

Key information in medicines guidelines may not be found and/or understood by healthcare professionals. User testing increased information retrieval and comprehension and could have an important role in improving the safety of medicines use.

## Abstract 2

Supplemental digital content is available in the text.

Approximately 237 million medication errors occur in England each year, of which 28% have potential to cause patient harm.^1^ The resultant additional inpatient admissions and increased hospital length of stay costs the National Health Service (NHS) at least £98 million per year.^1^ Internationally, the cost of unsafe medication practice and errors is estimated to be U.S. $42 billion.^2^ The risk of medication errors is higher for intravenous medicines than for other routes of administration, with 30% to 50% of intravenous doses involving an error.^3,4^ In 2017, the World Health Organization launched its third Global Patient Safety Challenge: Medication Without Harm, to reduce severe avoidable medication-related harm by 50% over 5 years.^2^

Written guidance for health professionals that is contradictory, incomprehensible, or of poor quality is one of many potential causes of medication errors.^5,6^ In particular, difficulty finding relevant, unambiguous information in technical documents has been linked to serious medication errors.^7–10^ There is little empirical evidence of the effectiveness of tools to prepare and test medicines guidelines for use by health professionals in the clinical environment. Only one study was identified, which used user testing with doctors to improve the presentation of the Summaries of Product Characteristics (SPCs) for 2 medicines, which increased the amount of information found and understood.^11^

User testing is based on the performance of a document in the hands of potential users, assessing whether people can find and understand the information they need.^12^ Problems and potential solutions are therefore identified. Iterative rounds of interviews with potential users are followed by document revision, until all issues are resolved.^11–13^ Each interview involves closed questions to determine whether the interviewee can locate and understand key information, followed by a semistructured interview to explore opinions of general issues. The validity of these opinions is enhanced by the fact that the participants have actually had to use the information (in a simulated situation).

User testing has been shown to improve understanding and decrease reading time of patient-facing medicines information, evidence summaries, and infection control guidelines.^12,14–16^ It is recommended for the implementation of clinical guidelines^17^ and is required for patient information leaflets for licensed medicines.^12^ However, because of lack of evidence, it is unclear whether user testing is effective in improving medicines guidance written for health professionals in the clinical environment.

Therefore, the aim of this study was to investigate the effectiveness of user testing for improving healthcare professionals’ retrieval and comprehension of information in medicines guidelines. The UK’s NHS Injectable Medicines Guide (IMG)^18^ was selected as a case study. This Web site provides guidance on the preparation and administration of intravenous medicines. In the United Kingdom, nurses prepare and administer most intravenous doses on hospital wards, and in more than 120 hospitals, they use the IMG as a source of information on how to do this. Where these hospitals use electronic prescribing and medicines administration systems, the IMG can also be integrated to give guidance from within the electronic system. However, 2 small surveys (1 of 145 hospitals across the United Kingdom, another of 11 hospitals in the southwest of England) have suggested the IMG is too detailed and confusing for some users.^19,20^ It is therefore a suitable case study for investigating the impact of user testing, especially given the increased risk of medication errors associated with intravenous administration.^3,4^ Therefore, the specific objective was to examine the effectiveness of user testing by nurses as a method of improving retrieval and comprehension of information from the IMG.

## METHODS

Three iterative rounds of user testing with hospital nurses were completed, each followed by document revision.^11–13^ To ensure that all aspects of the participants’ experiences were explored, this was underpinned by Rosenbaum’s user experience framework, which was developed to describe user experience of documents used in the development of evidence-based clinical guidelines.^21^ It describes 8 facets of a user’s experience of a document (Table 1). The design, implementation, and analysis of the study were informed by regular discussion with a multidisciplinary advisory group of hospital and community nurses, doctors, and pharmacists.

**TABLE 1:** The 8 Facets of Rosenbaum’s User Experience Framework^22^

Facet	Definition
Accessibility	Are there physical barriers to users gaining access to the document?
Findability	Can users locate what they are looking for?
Usefulness	Does the document have practical value for users?
Usability	How easy and satisfying is the document to use?
Understandability	This covers 2 types of comprehension: Do users understand what type of document they are looking at? Do users correct understand the content of the document in the way that the author intended?
Credibility	Is the document trustworthy?
Desirability	Is the document something the user wants or has a positive emotional response to?
Affiliation	Do users believe the document is intended to be used by “someone like me”?

### Participants

Included participants were nurses/midwives registered with the Nursing and Midwifery Council, who were authorized to prepare and administer intravenous medicines and had done so during at least 50% of shifts during the past 6 months. They were recruited from 3 NHS hospitals. At hospitals 1 and 2, the IMG was routinely used during intravenous medicines administration, but not at hospital 3.

Ten new participants took part in each round of user testing. This sample size is based on standard user testing methodology.^11–13^ As this is a form of diagnostic testing (establishing where documents do not work), a formal sample size calculation was not considered appropriate^13,22^; experience shows that most significant flaws are identified by the first few participants.^13^ Participants were purposefully sampled for each round to include a range of nursing experience (<5 and ≥5 years’ accreditation to prepare and administer intravenous medicines) and hospitals.

### Selection of Intravenous Medicines Guidelines for User Testing

Two current IMG guidelines were selected for testing: voriconazole and aminophylline. This selection was informed by a pilot study and input from the advisory group. Selection criteria were guidelines for high-risk medicines that collectively describe a variety of intravenous procedures, including reconstitution, dilution, and both short and continuous infusions. To minimize participants’ use of prior knowledge, the guidelines were anonymized by renaming the drugs “bathicillin” and “unimycin” (Supplementary File 1, http://links.lww.com/JPS/A329). The guidelines were presented to participants on a tablet computer.

### User Testing Procedure

User testing took place in a private, nonclinical setting. An interview schedule (Supplementary File 2, http://links.lww.com/JPS/A330) was prepared based on published user testing research,^11–13^ the Rosenbaum user experience framework.^21^ and the selected IMG guidelines. After introductory questions, each interview began with closed questions to determine whether the participant could locate and interpret 17 of the most important points of information in the IMG guidelines (Table 2). If necessary, the interviewer used nonleading prompts that did not assist participants in locating and interpreting information, such as repeating or clarifying the question. A range of question types were used to identify facts, appropriate actions, and explanations. Participants were alternately allocated to answer questions about voriconazole or aminophylline first. No time limit was applied. On completion of the closed questions, semistructured interviews (Supplementary File 2, http://links.lww.com/JPS/A330) were conducted to explore each nurse’s opinion of the content, design, and wording of the IMG guidelines. The interviews were audio recorded and field notes were made. Four pilot interviews were used to refine these techniques.

**TABLE 2:** Closed Questions About Voriconazole (“Bathicillin”) and Aminophylline (“Unimycin”)

1	How is intravenous bathicillin supplied?
2	Imagine you are reconstituting 200 mg of bathicillin. What do you need to use?
3	What infusion solutions can be used to dilute reconstituted bathicillin solution?
4	What should be monitored before starting a patient on bathicillin?
5	Suppose you are preparing a dose of 600 mg of bathicillin. What size infusion bag should be used?
6	How much sodium does the B-Cil brand of bathicillin contain?
7	Imagine you are making an infusion of 270 mg of bathicillin. How much reconstituted solution should you add to the infusion bag?
8	Can you give bathicillin to a patient who is allergic to latex?
9	Imagine you were giving a dose of 420 mg of bathicillin to a patient weighing 70 kg. What’s the shortest time the infusion should last?
10	In general, in what different ways can a dose of intravenous unimycin be given and why?
11	Round 1*: Suppose you were programming an infusion pump for a continuous infusion of unimycin at a rate of 700 μg/kg per hour for a patient weighing 65 kg. What infusion rate do you require in milliliter per hour? Rounds 2 and 3*: Suppose you were programming an infusion pump for a continuous infusion of unimycin at a rate of 45.5 mg/h. What infusion rate do you require in milliliters per hour?
12	What should you do if a patient develops low blood pressure while being given unimycin
13	Can you give an infusion of ciprofloxacin at the same time as an infusion of unimycin through the same cannula?
14	Suppose you prepare a continuous infusion of unimycin at 1 pm on Monday. What is the latest time you must stop using this infusion?
15	Why is extravasation of unimycin likely to be harmful?
16	Why is it important to double check the correct dose of unimycin has been prescribed?
17	What must you do if you need to give undiluted unimycin injection?

*As described in Table 5, after round 1, we revised the description of unimycin infusion rates in the guide, to ensure that it was aligned with users’ needs. This necessitated a change to question 11.

### Analysis and Guideline Revision

The number of participants in each round able to both find and understand each of the 17 important points of information was determined by scoring against prespecified criteria (Supplementary File 2, http://links.lww.com/JPS/A330). A response was scored “found with ease” if the participant located at least part of the required information. If the participant took more than 60 seconds or required 2 or more prompts, it was scored “found with difficulty.” If a participant did not locate any of the required information, the response was scored “not found.” Responses were scored “understood” if the participant interpreted the information located to give the complete prespecified answer. Partial answers and incorrect calculations were scored “not understood.” Where a response was scored “not found,” understanding was scored as “not applicable.”

Interview recordings were transcribed and anonymized, then analyzed using thematic analysis based on the 6 stages described by Braun and Clarke.^23^ The aim was to produce a detailed account of views that would help improve the performance of the IMG. A mixed inductive and deductive approach using Rosenbaum’s user experience framework^21^ was used to ensure that all aspects of participants’ experience of the IMG were explored. Transcripts were read and potential emerging codes were noted. Transcripts were coded in Nvivo (Version 11; QSR International). Codes were reviewed and sorted into the 8 facets of the Rosenbaum user experience framework (Table 1), while also grouping codes that did not fit the framework into emerging themes. Codes and themes were refined iteratively to produce a descriptive account of the data.

After each round of user testing, the IMG guidelines were revised based on the participants’ responses and information design best practice.^24–26^ To ensure acceptability, changes were discussed with the advisory group. Revised guidelines were tested in the subsequent round.

### Ethics Approval

This study was approved by the University of Bath Research Ethics Approvals Committee for Health (Reference Number EP17/18-126) and the NHS Health Research Authority (IRAS Number 235214).

## RESULTS

Thirty nurses with experience in a wide range of clinical areas participated (Table 3).

**TABLE 3:** Participant Characteristics (n = 30 Overall, n = 10 in Each Round)

	No. Female Participants	Median Age (Interquartile Range)	No. Participants From Each Hospital			Median Years Nursing Experience (Interquartile Range)	Median Percentage of Shifts in Which Intravenous Medicines Administered (Interquartile Range)	Number With English as First Language
			1	2	3			
Round 1	9/10	32 (25–48)	5	2	3	7 (3–12)	100 (88–100)	7/10
Round 2	8/10	32 (26–45)	2	6	2	5 (3–9)	100 (98–100)	9/10
Round 3	10/10	38 (25–44)	2	3	5	7 (2–19)	100 (94–100)	8/10
Overall	27/30	33 (26–45)	9	11	10	6 (3–12)	100 (94–100)	24/30

### Round 1 Closed Questions and Guideline Revisions

In round 1, fewer than half of the closed questions were answered correctly by all participants (Table 4). Question (Q) 7 (relating to voriconazole dose measurement) and Q12 (aminophylline adverse effects) were the questions for which information was most often not found. For Q7, many participants did not locate the voriconazole displacement volume and thus used an incorrect drug concentration. Question 5 (voriconazole infusion bag size), Q9 (voriconazole infusion time), and Q11 (aminophylline infusion rate) had the most commonly misunderstood information, all of which were related to calculation errors.

**TABLE 4:** Number of Participants in Each Round Scored as Finding and Understanding the Information Needed to Answer Each of the 17 Closed User Testing Questions

Question	Information Found With Ease*			Information Found With Difficulty^†^			Found and Understood^‡^		
	Round 1 (n = 10)	Round 2 (n = 10)	Round 3 (n = 10)	Round 1 (n = 10)	Round 2 (n = 10)	Round 3 (n = 10)	Round 1^§^ (n = 10)	Round 2^§^ (n = 10)	Round 3^§^ (n = 10)
1	10	10	10	0	0	0	10	10	10
2	9	10	10	1	0	0	10	10	10
3	10	10	10	0	0	0	10	10	10
4	5	8	10	4	2	0	9	10	10
5	1	10	10	6	0	0	3	10	10
6	10	9	10	0	1	0	10	10	10
7	2	4	5	1	1	4	3	4	8
8	9	10	10	1	0	0	10	10	10
9	5	9	9	5	1	1	6	9	9
10	10	10	10	0	0	0	8	10	10
11	3	6	5	7	4	5	7	10	10
12	2	10	10	2	0	0	4	10	10
13	10	10	10	0	0	0	10	10	10
14	9	9	7	1	1	3	10	10	10
15	10	10	10	0	0	0	9	10	10
16	5	7	9	1	2	1	5	9	10
17	5	6	6	5	4	4	10	10	10

*As described in the methods section, a response was scored as “found with ease” if the participant located at least part of the required information in less than 60 seconds and with fewer than 2 prompts.

^†^As described in the methods section, a response was scored as “found with difficulty” if the participant located the first part of the required information in more than 60 seconds or with 2 or more prompts. In the results, all participants scored as finding information “with difficulty” took more than 60 seconds to locate the required information, although 8 participants also required 2 prompts.

^‡^As described in the methods section, responses were scored as “understood” if the participant interpreted the information located to give the complete prespecified answer. Data are presented for the number of participants finding and understanding, as finding information is a prerequisite of understanding it, so participants who could not find information were scored “not applicable” for understanding.

^§^As there were 10 participants in each round, a number less than 10 indicates that some participants were unable to find and understand the required information.

Numerous changes were made to the wording, structure, and design of the IMG guidelines (Supplementary File 3, http://links.lww.com/JPS/A331) to address the problems identified through both the closed questions and the semistructured interviews. Removal of unnecessary words, and addition of bullet points, active voice, and bold text for emphasis (instead of capitals) were among the changes made. Revisions related to participants’ difficulties with calculations are summarized in Table 5.

**TABLE 5:** Participants’ Problems With Calculations Identified During Round 1 and the Consequent Guide Revisions Made Before Round 2

Direct Question	Round 1 Problem	Guide Revision Before Round 2
Q5: Suppose you are preparing a dose of 600 mg of bathicillin. What size infusion bag should be used?	Three participants did not find the voriconazole concentration range required to answer this question. Four further participants could not use this information to calculate the correct answer	Provide suggested dilution volumes instead of a concentration range, thus removing the need for a calculation
Q7: Imagine you are making an infusion of 270 mg of bathicillin. How much reconstituted solution should you add to the infusion bag?	Seven participants did not account for the displacement volume of voriconazole	Use bold text and bullet points to emphasize the concentration of the reconstituted solution.
Q9: Imagine you were giving a dose of 420 mg of bathicillin to a patient weighing 70 kg. What’s the shortest time the infusion should last?	Four participants could not perform the calculation needed to answer this question	Provide an equation and table to calculate the infusion rate
Q11: Suppose you were programming an infusion pump for a continuous infusion of unimycin at a rate of 700 μg/kg per hour for a patient weighing 65 kg. What infusion rate do you require in milliliter per hour?	Participants stated that weight-based infusion rate calculations were not relevant to their practice, as weight-based calculations were performed by prescribers	Move this information to a new “example dose calculation” subsection, and add an equation and example calculation linking infusion rate, prescribed dose and concentration

Participants were observed to use subsection titles to locate the information they required. Therefore, subsection titles were changed from capitals to sentence case to improve readability^24^ and highlighted with a page-width colored bar to ensure clear differentiation between subsections. In addition, a number of new subsections were introduced. Participants found it difficult to find information on pretreatment checks for both medicines (Q4 and Q16) as this information was not presented at the start of each guideline, where they said they expected to find it. A “Before Treatment” subsection was therefore created. A new “Administration” subsection was also created, to avoid the need to scroll from the preparation information to the top of the document for administration guidance. Participants requested a summary of key information for each medicine, as detailed information is not required for most patients. Therefore, a “Summary” was added to the start of each guideline.

Advice on managing acute adverse effects of aminophylline was moved from the “method of administration” subsection to the “adverse effects and monitoring” subsection, where participants expected it to be located (Q12).

### Round 2 Closed Questions and Guideline Revisions

The number of participants able to find and understand the information required increased when the revised monographs were tested in round 2 (Table 4). Information remained not found most often for Q7 (voriconazole dose measurement), although more people found the necessary information than in round 1. Once again, this was because participants did not account for the displacement volume. The only closed questions for which information was misunderstood were Q7 and Q9 (voriconazole infusion time). In each case, one participant made a calculation error.

After completion of round 2, fewer revisions were required (Supplementary File 4, http://links.lww.com/JPS/A332). To address the problems related to Q7 (voriconazole dose measurement), the description of the concentration of reconstituted voriconazole solution was moved into the same bullet as the description of reconstitution, as many round 2 participants did not read as far as the concentration description. Several participants stated that they did not recognize the concentration description (“10 mg in 1 mL”) as relevant, as they were considering 200 mg reconstituted with 19 mL. Therefore, the description of this concentration was changed to “20 mL containing 200 mg.” To address the problems with Q9 (voriconazole infusion time), the link to the table of infusion times was made more prominent. Finally, the “summary” subsection added at the end of round 1 was removed, because most participants stated that there were too many subheadings and they would not read beyond the summary, so they would be concerned that they would not read potentially important advice. In addition, the summary attracted attention, so participants reported being less likely to notice smaller subsections nearby. In place of the summary, darker bars for the “preparation” and “administration” subsection headings were introduced, to alert users to the location of this key information.

### Round 3 Closed Questions and Guideline Revisions

The guidelines tested in round 3 performed better overall than those tested in round 2 (Table 4). More participants were able to find and understand the information required. Question 7 (voriconazole dose measurement) was still the closed question for which the fewest participants (8 of 10) found and understood the required information, but this was improved compared with round 2.

Limited changes were made to the IMG guidelines after round 3 (Supplementary File 5, http://links.lww.com/JPS/A333). An equation for calculating the required volume of reconstituted voriconazole solution was added, as one participant had located the drug concentration but was unsure how to use it, while another had not accounted for the displacement volume. To ensure familiarity to typical users, this equation was written in the form described by many participants.

### Overall Performance of the Guidelines

**FIGURE 1:**
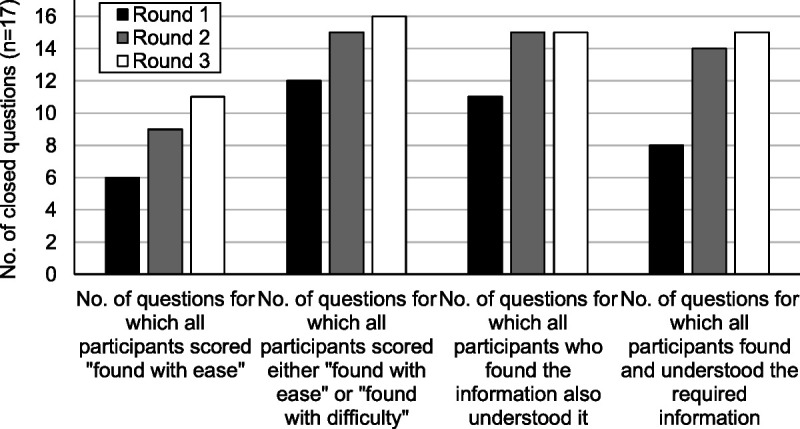
The number of closed questions (n = 17) in each round of user testing which achieved various measures of overall performance. As described in the methods section, a response was scored as “found with ease” if the participant located at least part of the required information. If the participant took more than 60 seconds or required 2 or more prompts, it was scored “found with difficulty.” Responses were scored as “understood” if the participant interpreted the information located to give the complete prespecified answer.

Figure 1 summarizes the overall performance of the IMG guidelines and shows that by identifying problems with the IMG guidelines and introducing revised designs, the user testing process improved their performance. During round 1 there were 36 cases (of a potential 340) of a participant being unable to find or understand an important point of information, compared with only 3 cases during round 3.

### Participants’ Opinions of Content, Design, and Wording

Codes relating to all 8 facets of Rosenbaum’s user experience framework were identified. The 6 facets most relevant to the aims of this study are described hereinafter.

### Facet 1: Affiliation

Participants in all rounds clearly felt that the guidelines tested were intended to be used by someone performing their professional role. This especially applied to the first half of each guideline (Table 6, quotation 1). Two reasons were given: the content was relevant to the tasks they had to perform and the language was appropriate. However, some round 1 participants also suggested that the guidelines were intended to be used by someone with more reading time than is typically available (Table 6, quotation 2). The absence of similar comments in later rounds may suggest that the revisions were successful in this respect.

**TABLE 6:** Selected Quotations From the Semi-Structured Interviews

	Quotation (Participant Number)	Round	Sex	Years of Experience	Hospital
1	“I think so, I think definitely the first part would definitely be aimed at a nurse who was preparing and administering these. As I said before there is parts of the second part that I just do not think would be relative to nursing staff.” (A19)	2	Female	3	2
2	“… they are presuming you have half an hour to read all this before you are dishing the drug out which in an ideal world you would…” (A8)	1	Female	2.5	1
3	“… and use of the big blue lines is breaking it down to bits that I need. I think it will be easier to click through the screen and find the information that I need simply because it seems separated.” (A18)	2	Male	4.5	2
4	“… there is preparation and administration isn't there, there is the 2 different parts of it, you prep a drug and then you go and administer it so I suppose there, even though it is good having the times at the top you almost want your preparation bit first and then your method of actually administering and the prep would be the reconstitution and then which bag to put it in.” (A11)	1	Female	9	1
5	“… it is laid out in a way that it’s a natural progression of this is what I want, how am I making it, how am I giving it?” (A33)	3	Female	2	1
6	“… I think if you read the whole thing from start to finish all the information is there isn't it but no one does that, you skip down for the heading you are looking for don't you.” (A11)	1	Female	9	1
7	“It’s there, sometimes I feel it’s not worded the right way and I confuse myself and I have to double check with colleagues or pharmacy, but it is there.” (A5)	1	Female	5	1
8	“It is quite clear, it is very specific which is good.” (A15)	2	Female	3	2
9	“… there is too many words and it’s too complicated. Which when you are giving IVs you want a very simple and you want it very obvious, if there is this amount of writing…it can take you 20 minutes to actually find all the information, that’s where your errors hop in, because it’s not obvious.” (A13)	1	Female	31	3
10	“There always seems to be a lot of writing but that is because there is a lot of information that has got to be put over” (A8)	1	Female	3	1
11	“They were really useful, they are probably the most in depth prompt I have seen. Normally we just get a page of the basics… but it has got all the information and it is quite clearly laid out from…the order you would do it in.” (A31)	3	Female	2	3

### Facet 2: Credibility

Participants identified a range of IMG features affecting credibility. These included lists of references and suppliers, as well as comprehensive and clear content in rounds 2 and 3. A number of features suggested in round 1 were introduced in round 2 (e.g., prominent publication date and NHS branding), where participants commented that they increased credibility.

### Facet 3: Findability

Across all 3 rounds, participants commented that highlighting key information with bold text and subheadings increased findability. Many participants found the revised subheading design in rounds 2 and 3 aided findability (Table 6, quotation 3).

Participants expected the guidelines to be presented in the order in which the information is needed while preparing a medicine. Participants in round 1 described various problems with the order of the information (Table 6, quotation 4). Some participants in all rounds felt that the information was presented in the right order, but this became progressively more common through the 3 rounds (Table 6, quotation 5).

Participants in all rounds described using a search strategy based on subheadings (Table 6, quotation 6). However, participants in round 1 found that they needed to scroll through a lot of information to find what they needed. This was viewed negatively (as it caused delay) and was linked to difficulty knowing where to find certain information. A range of specific types of information were described as easy to find across all the rounds. Conversely, some types of information were described as hard to find, including adverse effects, the “before treatment” subsection and drug concentrations. These comments were mainly in round 1.

### Facet 4: Understandability

In round 1, participants commented that although they understood most of the content, some of it could be improved and they sometimes needed help from a colleague (Table 6, quotation 7). More in rounds 2 and 3 thought that the content of the guidelines was easy to understand. Participants suggested that specific, detailed, and practical instructions helped make guidelines more understandable, while excessive wordiness made them less understandable (Table 6, quotations 8 and 9).

### Facet 5: Usability

Participants in all rounds, especially rounds 2 and 3, commented that the guidelines were generally clear and easy to use. However, some in rounds 1 and 2 held more nuanced opinions: although the guidelines were usable, this could be improved, or that the guidelines were easy to use once they were familiar. Some participants thought that the guidelines presented an overwhelming amount of information; however, there was recognition that this was necessary (Table 6, quotation 10).

### Facet 6: Usefulness

Participants (especially in rounds 2 and 3) thought that the guidelines would be of practical use in their daily practice. However, some participants in round 1 and one in round 2 commented that although the guidelines were useful, this could be improved. Many participants in all rounds commented that the guidelines were useful because they provided comprehensive information and specific detail (Table 6, quotation 11). Participants in all rounds commented on the usefulness of the different types of information contained within the guidelines. Some participants commented that all the information would be useful. Many types of information were highlighted as being particularly important or unimportant with considerable overlap between these 2 conflicting lists.

Participants in rounds 2 and 3 commented that it was easier to find information in the guideline they tested and that it was more understandable and usable, compared with the current IMG.

## DISCUSSION

This study reports only the second application of user testing as a tool to improve the retrieval and comprehension of written medicines information for health professionals. It is the first such study of guidelines for nurses about safe medicines administration. Despite the current use of the IMG in everyday practice, the results demonstrated that many practicing nurses were unable to find and/or understand important points of information. By identifying these problems and potential solutions, user testing improved the performance of the guidelines, such that by round 3, there were only 3 instances of a participant being unable to find or understand an important point of information.

The results of this study are comparable with those of Raynor et al,^11^ who applied user testing with doctors to improve the presentation of SPCs for 2 medicines. Both studies resulted in similar changes to the information under investigation (e.g., use of bullet points and bold text, new subsections, and moving information to make it easier to find). Information retrieval and comprehension improved after user testing in both studies; however, performance of the final documents in the current study was better than that of Raynor et al.^11^ This may be because SPCs are longer documents that cover a greater range of complex topics. This could also explain why a summary section was deemed useful by Raynor et al,^11^ but not in the present study. Similarly, the application of user testing to nonmedicines guidance for health professionals has also resulted in improved document performance by resolving similar difficulties to those identified in this study (e.g., lack of specific detail and difficulty interpreting numerical information).^14–16^

To the best of our knowledge, this is the first study to examine the application of user testing to improve the performance of medicines guidance for nurses. Among other strengths of this study are the use of new participants in each round and guideline anonymization, to minimize learning effects. However, the principal limitation of this study is that the performance of the IMG guidelines was tested in an interview rather than in clinical practice. Therefore, the next stage of our research will be to find out whether use of the revised guidelines results in fewer errors in preparation and administration of intravenous medicines in a clinical environment. In addition, the IMG includes information on hundreds of medicines, so it is unclear whether these findings would be replicated with other medicines, such as those that are simpler to prepare and administer. However, many of the revisions introduced in this study (e.g., subsection title design, new subsections, increased support for calculations) could be applied to any medicine in the IMG.

This study has demonstrated that key points of information in medicines guidelines may not be found and understood by health professionals, even when they have been written using a quality assurance process to ensure accuracy and comprehensiveness.^18^ As the points of information that were not found and/or understood in this study related to key steps in the preparation and administration of intravenous medicines (e.g., measuring the correct dose and infusing it at the right rate), such problems with the design of guidelines might increase the risk of serious errors. Authors should therefore ensure that healthcare professional medicines guidelines are usable for their target readers, as well as being accurate and comprehensive. User testing can help achieve this, as it improved the performance of the guidelines and may therefore increase medicines safety by improving the quality of professional guidelines, a recognized cause of medication errors.^5,6^ User tested patient medicines information is a requirement for licensed medicines.^12^ An additional requirement for manufacturers to supply user tested professional medicines information in a standard format might reduce the need for healthcare providers to produce bespoke guidance such as the IMG.

As the effectiveness of user testing medicines guidelines for health professionals has been examined only twice, future research should test different types of medicines information (e.g., the British National Formulary or locally produced guidelines) and groups of health professionals (e.g., pharmacists).

## CONCLUSIONS

Healthcare professionals may not be able to find and understand key topics in written medicines guidelines used in everyday practice. However, user testing can improve both the retrieval and comprehension of information.

## Supplementary Material

SUPPLEMENTARY MATERIAL
