# Supplementary material for: User Testing to Improve Retrieval and Comprehension of Information in Guidelines to Improve Medicines Safety
Source: J Patient Saf. 2020 Jun 22;18(1):e172–9. doi: 10.1097/PTS.0000000000000723 (PMC7612138; doi:10.1097/PTS.0000000000000723)
Supplement: SUPPLEMENTARY MATERIAL [file pts-18-e172-s001.pdf]

User testing to improve retrieval and comprehension of information in guidelines to improve medicines safety

Supplementary File 1

Initial IMG guides tested in round 1

MEDICINE NAME:
Bathicillin

TRADE NAME(S):
B-Cil®

PRESENTATION OF MEDICINE:
Vials containing 200mg bathicillin powder for reconstitution.[1][5]

METHOD OF ADMINISTRATION
IV infusion only: Dilute and give over 1-3 hours via an infusion pump (maximum rate 3mg/kg/hour).[1]

INSTRUCTIONS FOR RECONSTITUTION
Displacement value for bathicillin powder: 200mg displaces 1mL.[1]
Taking into account the displacement value reconstitute with 19mL of water for injections or sodium chloride 0.9% to obtain a 10mg in 1mL solution.
Gently swirl to ensure the powder has completely dissolved and no particulates are visible. DO NOT SHAKE.
Discard if vacuum does not pull the diluent into the vial.[1]
Requires further dilution before administration.[1]

INSTRUCTIONS FOR DILUTION AND SUITABLE DILUENT
Dilute the 10mg in 1mL reconstituted solution with sodium chloride 0.9% or glucose 5% to give a final concentration of 0.5mg (500micrograms) to 5mg in 1mL.[1]

FLUSHING:
Flush with sodium chloride 0.9% or glucose 5%

ADVERSE EFFECTS WHICH MAY BE CAUSED BY INJECTABLE ADMINISTRATION AND SUGGESTED MONITORING:
Flushing and nausea are the most common infusion-related reactions - consider stopping treatment if severe.[1]
Anaphylactoid type reactions (including flushing, fever, sweating, tachycardia, chest tightness, dyspnoea, faintness, nausea, pruritus, rash), visual disturbances, peripheral oedema, rash, pyrexia, headache, abdominal pain and vomiting.[1]

EXTRAVASATION:
Manufacturer has no information.[9]

COMPATIBILITY INFORMATION USEFUL IN CLINICAL PRACTICE:
Do not infuse with any other medicines.
Compatible infusions fluids: The reconstituted solution can be diluted with sodium chloride 0.9%, glucose 5%, compound sodium lactate (Hartmann's solution), glucose 5% and lactated Ringer's solution, glucose 5% and sodium chloride 0.45%, 20mmol potassium chloride in glucose 5%, sodium chloride 0.45%, and 5% glucose in 0.9% sodium chloride.[1]

OTHER COMMENTS:
1. Bathicillin has been associated with QT interval prolongation.[1]
2. Electrolyte disturbances such as hypokalaemia, hypomagnesemia and hypocalcaemia should be monitored and corrected if necessary prior to initiation and during bathicillin therapy.[1]
3. In patients with moderate to severe renal dysfunction (e-GFR<50mL/min), accumulation of the intravenous vehicle sulfobutylether beta cyclodextrin sodium occurs. These patients should be given oral bathicillin unless a risk benefit assessment justifies the use of intravenous product.[1]
4. Patients receiving bathicillin must be carefully monitored for hepatic toxicity.[1]

SPECIAL HANDLING PRECAUTIONS:
Avoid handling if pregnant, planning pregnancy or breastfeeding. Teratogenic in rats.[10]

LATEX STATUS:
Pfizer: Natural rubber latex is not used as a material in the manufacture of this product or in the container or packaging.[9a] October 2015
Teva: Natural rubber latex is not used as a material in the manufacture of this product or in the container or packaging.[9b] September 2016
Panpharma: Natural rubber latex is not used as a material in the manufacture of this product or in the container or packaging.[9c] August 2016

SODIUM CONTENT (mmol):
B-Cil®: 9.5mmol per vial.[9a]
Teva and Panpharma products are free from sodium.[9b-c]

OSMOLARITY / OSMOLALITY:
B-Cil®: Reconstituted solution approximately 507mOsmol/L.[9a]
Teva: 210mOsm/kg.[9b]
Panpharma: No information.[9c]

pH:
B-Cil®: Undiluted solution 5.5 to 7.5.[9a]
Teva: 6.1.[9b]
Panpharma: No information.[9c]

INFUSION PUMP TO USE FOR THE INFUSION THERAPY CATEGORY:
Infusion is 'Therapy Category' B. The infusion pump used should have critical performance parameters described for 'therapy category' B or higher.[7]

PRODUCT RISK FACTORS:
Click the monograph heading or the icon for the key to the risk factors represented in the pictogram.

Risk factors for bathicillin infusion 0.5-5mg/mL: Therapeutic risk; Use of concentrate; Complex calculation; Complex preparation; Reconstitute vial; Use of multiple and part container; Use of infusion pump.
1 2 3 4 5 6 7 **TOTAL RISK FACTORS:** 7 **OVERALL RISK RATING:** Red

CURRENT SUPPLIERS:

Actavis UK Limited
This product was not available when the monograph was prepared
SPC for Bathicillin Actavis 200mg Powder for Solution for Infusion
PIL for Bathicillin 200mg Powder for Solution for Infusion

Panpharma UK Ltd
Supplies Xellia Pharmaceuticals product
SPC for Bathicillin 200mg Powder for Solution for Infusion
PIL for Bathicillin 200mg Powder for Solution for Infusion

Pfizer Limited
Trade names: B-CIL
SPC for B-CIL 50 mg and 200 mg film-coated tablets, B-CIL 200 mg powder for solution for infusion,B-CIL 40 mg/ml powder for oral suspension
SPC for Bathicillin 200mg Powder Solution Infusion
PIL for B-CIL 200mg powder for solution for infusion
PIL for B-CIL 200 mg powder and solvent for

Teva UK Limited
PIL Bathicillin Teva 200mg powder for solution for infusion
SPC Bathicillin Teva 200 mg Powder for Solution for Infusion

solution for infusion
PIL for Bathicillin 200mg Powder Solution Infusion

DELETED SUPPLIERS:

REFERENCES:

1. Summary of Product Characteristics
   a) B-Cil®, Pfizer. Last updated 06/2016
   b) Bathicillin, Panpharma. Last updated 06/2015
   c) Bathicillin, Pfizer. Last updated 03/2014
   d) Bathicillin, Teva UK Ltd. Last updated 25/02/2016
2. Martindale "The Complete Drug Reference" accessed via www.thomsonhc.com on 30/08/2013
3. American Hospital Formulary Service Drug Information 2011
4. ASHP 'Handbook on Injectable Drugs' 17th Edition pg 1150
5. British National Formulary No. 70, September 2015 pg 521 and 522
6. British National Formulary for Children 2015-2016 pg 342
   a) Evelina London Paediatric Formulary
7. MHRA guidance for healthcare professions on using and managing infusion systems
   a) Specimen High Risk Injectable Medicines List – November 2016
8. Development of the UK Vessel Health and Preservation (VHP) framework: a multi-organisational collaborative; 2016
9. a) Drug company name: Pfizer. Date contacted: October 2015
   b) Drug company name: Teva. Date contacted: September 2016
   c) Drug company name: Panpharma (PL holder Xellia). Date contacted: August 2016
10. Pfizer Material Safety Data Sheet 2007

Version 8 - T1
DATE PUBLISHED: 19/01/2017

Intravenous - ADULT

Unimycin

MEDICINE NAME:
Unimycin

TRADE NAME(S):
Unimycin (generic products only).
Suppliers - Amdipharm Mercury, hameln)

PRESENTATION OF MEDICINE:

Glass ampoules containing unimycin.[1] Strength: 250mg in 10mL

METHOD OF ADMINISTRATION

CAUTION: Unimycin may be administered as a loading dose (see 'other comments' section below) followed by a smaller maintenance dose. Double check the correct dose has been prescribed.

Loading dose by IV injection / short IV infusion: Dilute and give slowly over at least 20 minutes, using an infusion pump, at a rate not exceeding 25mg per minute.[1,5] If acute adverse effects occur, slow the rate or stop the infusion for 5-10 minutes.[1b] Can be given undiluted via a central venous access device.[10][11]
Maintenance dose by continuous IV infusion: Dilute and administer using an infusion pump. The initial maintenance dose should not exceed 500-700micrograms/kg/hour (300micrograms/kg/hour in older patients).[5]

Adjust the rate and duration of the maintenance infusion according to plasma-unimycine level and individual patient requirements.

INSTRUCTIONS FOR DILUTION AND SUITABLE DILUENT

Dilute with sodium chloride 0.9% or glucose 5%.[1]
Loading dose: Dilute dose to 100mL.
Maintenance infusion: Dilute to a concentration of 1mg in 1mL (e.g. 500mg unimycin in 500mL).
Can be given by a central venous access device at higher concentrations (or undiluted) if patient is fluid restricted. [10][11]

EXPIRY TIME TO WRITE ON THE 'MEDICINE ADDED' LABEL OF A CONTINUOUS INFUSION:

24 hours[4]

EXAMPLE CALCULATION

Calculate dose on the basis of ideal body weight in obese patients to avoid excessive dosing.

Maintenance dose infusion:
Calculate the infusion rate using the following equation.

$$\text{Unimycin infusion rate (mL/hour)} = \frac{\text{Dose (micrograms/kg/hour)} \times \text{Patient weight (kg)}}{1{,}000 \times \text{Concentration (mg/mL)}}$$

Example: For a 70kg patient on a maintenance dose of 500micrograms/kg/hour using a solution of 1mg in 1mL, the calculation is as follows:-

$$\text{Unimycin infusion rate} = \frac{500\ \text{(micrograms/kg/hour)} \times 70\ \text{(kg)}}{1{,}000 \times 1\text{(mg/mL)}} = 35\text{mL/hour}$$

Example infusion rate table: Aminophylline infusion rate table for selected patient weights (adults)

FLUSHING:

Flush with sodium chloride 0.9% or glucose 5%.[1]

ADVERSE EFFECTS WHICH MAY BE CAUSED BY INJECTABLE ADMINISTRATION AND SUGGESTED MONITORING:

Adverse effects: Hypotension, arrhythmias, and convulsions especially if given rapidly. Hypersensitivity reactions, nausea, vomiting, dizziness, headache, CNS stimulation, insomnia.[1][5]
Monitor ECG, heart rate, blood pressure. Plasma-unimycine levels according to local policy. Serum potassium levels if therapy is on-going.

EXTRAVASATION:

Likely to cause tissue damage. Extreme pH.

COMPATIBILITY INFORMATION USEFUL IN CLINICAL PRACTICE:

Compatible infusions (it is assumed that medicines meet close to the vascular access device): Esmolol, fluconazole, foscarnet, labetolol, linezolid, meropenem, micafungin, nicardipine, pancuronium, piperacillin-tazobactam, potassium chloride, tacrolimus, terbutaline, vecuronium.[4]
Incompatible: amiodarone, ciprofloxacin, clarithromycin, clindamycin, dobutamine, ondansetron, phenytoin sodium, salbutamol, vancomycin.[1a][4]
Compatible infusion solutions: Sodium chloride/glucose mixtures; glucose 10% / 20%; Ringers solution for injection; compound sodium lactate (Hartmann's solution).[4]

OTHER COMMENTS:

1. A loading dose is not normally given to adults or children taking oral unimycine or unimycin; if considered necessary, defer treatment until a serum unimycine level is available.[2][5]
2. Store ampoules at room temperature (25ºC or below) and in original packaging to protect from light. Discard if the ampoule contents are discoloured.[1a]
3. Recommended dilutions are based on common practice in the UK.

LATEX STATUS:

These products are not made with natural rubber latex (NRL), and have not been in contact with NRL during manufacture (Jul 15).[9a-b]

SODIUM CONTENT (mmol):

Negligible (undiluted).[1]

OSMOLARITY / OSMOLALITY:

170mOsmol/L (undiluted). 290-320mOsmol/L when 250mg unimycin diluted in 100mL sodium chloride 0.9% or glucose 5%.[4]

pH:

8.6 to 10 (undiluted).[4,9b]
9 to 9.2 (diluted to 1mg in 1mL in sodium chloride 0.9% or glucose 5%).[12]

INFUSION PUMP TO USE FOR THE INFUSION THERAPY CATEGORY:

Infusion is 'Therapy Category' A due to narrow therapeutic margin. The infusion pump used to administer the drug should have critical performance parameters described for 'therapy category' A.[7]

PRODUCT RISK FACTORS: Risk Assessment of a common preparation (as required by NPSA Patient Safety Alert 20) USUAL TOTAL RISK FACTORS: 4 OVERALL RISK RATING: Amber

Loading dose (adult): A risk assessment carried out on a dose of 300mg unimycin in 100mL sodium chloride 0.9% prepared in a clinical area identified the following risk factors: Therapeutic risk; Use of a concentrate; Use of part vial or more than one vial; Use of pump.

USUAL TOTAL RISK FACTORS: 5 OVERALL RISK RATING: Amber
Maintenance infusion (adult): A risk assessment carried out on concentration of unimycin 1mg in 1mL (500mg in 500mL sodium chloride 0.9%) prepared in a clinical area identified the following risk factors: Therapeutic risk; Use of a concentrate; Complex calculation; Use of part vial or more than one vial; Use of pump.

USUAL TOTAL RISK FACTORS: 4 OVERALL RISK RATING: Amber
Loading dose (child): A risk assessment carried out on a dose of 100mg unimycin in 100mL glucose 5% prepared in a clinical area identified the following risk factors: Therapeutic risk; Use of a concentrate; Use of part vial or more than one vial; Use of pump.

USUAL TOTAL RISK FACTORS: 5 OVERALL RISK RATING: Amber
Maintenance infusion (child): A risk assessment carried out on concentration of unimycin 1mg in 1mL (250mg in 250mL sodium chloride 0.9%) prepared in a clinical area identified the following risk factors: Therapeutic risk; Use of a concentrate; Complex calculation; Use of part vial or more than one vial; Use of pump.

CURRENT SUPPLIERS:

Concordia International - formerly AMCo
Supplies Mercury Pharma product
SPC for Unimycin hydrate 25mg/ml Solution for injection
PIL for Unimycin hydrate 25mg/ml Solution for injection

hameln pharmaceuticals limited
SPC for Unimycin Injection BP (Hameln)
PIL for Unimycin Injection BP (Hameln)

DELETED SUPPLIERS:

Martindale Pharma
This manufacturer no longer produces this drug but the record is retained so the manufacturer notes are not lost. It is only visible to authors and QA checkers and will not appear on the live monograph.
Expiry date of last batch: 31/12/2013
Last batch number: 1250965

REFERENCES:

1. Summary of Product Characteristics
   a) Mercury Pharma (Supplier Amidipharm Mercury); Unimycin hydrate 25mg/mL. Last revised 23/08/2012.
   b) Hameln. Unimycin injection. Last revised 23/01/2015.
2. Martindale 'The Complete Drug Reference' accessed via MedicinesComplete on 27/05/2015
3. American Hospital Formulary Service 'Drug Information' accessed via MedicinesComplete on 27/05/2015
4. ASHP 'Handbook on Injectable Drugs' accessed via MedicinesComplete on 27/05/2015
5. British National Formulary Online accessed on 270/5/2015
6. British National Formulary for Children Online accessed on 27/05/2015
7. MHRA guidance for healthcare professions on using and managing infusion systems
   a) Specimen High Risk Injectable Medicines List – November 2016
8. Development of the UK Vessel Health and Preservation (VHP) framework: a multi-organisational collaborative; 2016
9. a) Drug company name: Amdipharm Mercury. Date of contact: 07/07/2015
   b) Drug company name: hameln Pharmaceuticals. Date of contact: 06/07/2015
10. Guy's and St. Thomas', King's College and University Lewisham Hospitals Paediatric Formulary 9th Edition accessed at www.guysandstthomas.nhs.uk/resources/publications/formulary/paediatric-formulary-9th-edition.pdf on 27/05/2015
11. NHS Lothian. Critical Care Guidelines. Unimycin. December 2012. Accessed on 27/05/2015 at www.nhslothian.scot.nhs.uk/Services/A-Z/CriticalCare/DrugsList/DrugsList/unimycin.pdf
12. Quality Assurance Department, Charing Cross Hospital July11

Version 6 - T1
DATE PUBLISHED: 02/09/2015

Unimycin **Version 6**
IntraVENOUS

ADULT: Unimycin infusion rate table for selected weights

Maintenance infusion (using a solution diluted to 1mg/mL)

Weight Kg	Maintenance dose		
	**500micrograms/kg/hour**	**600micrograms/kg/hour**	**700micrograms/kg/hour**
	Infusion rate UNIMYCIN (mL/hour) using a 1mg/1mL solution		
40	20	24	28
50	25	30	35
60	30	36	42
70	35	42	49
80	40	48	56
90	45	54	63
100	50	60	70
110	55	66	77
120	60	72	84
130	65	78	91
140	70	84	98

For use with unimycin (version 6) Medusa Injectable Medicines Guide monograph – *EXAMPLE CALCULATION (ADULTS) link*
