# Supplementary material for: User Testing to Improve Retrieval and Comprehension of Information in Guidelines to Improve Medicines Safety
Source: J Patient Saf. 2020 Jun 22;18(1):e172–9. doi: 10.1097/PTS.0000000000000723 (PMC7612138; doi:10.1097/PTS.0000000000000723)
Supplement: SUPPLEMENTARY MATERIAL [file pts-18-e172-s002.pdf]

User testing to improve retrieval and comprehension of information in guidelines to improve medicines safety

Supplementary File 2

Interview schedules and pre-specified answer criteria:

- Round 1
- Rounds 2 and 3

Interview schedule – researcher (round 1)

Effects of User Testing Injectable Medicines Guidance – Phase 1

Thank you for taking the time to speak to me today. Your help with this study is greatly appreciated.

This interview should last about 45-60 minutes, although if you would like to pause at any point, please let me know. If you decide that you no longer want to take part, please also let me know, and we can stop the interview.

The interview will have three parts. In the first part, I will ask you a few questions about your experience of preparing and administering intravenous medicines. Then I will give you two intravenous administration guides for invented medicine called "bathicillin" and "unimycin". I will ask you questions about these medicines and ask you to answer them by finding the information in the guides. In the final part of the interview, I will ask you some questions about how you found using the guide.

It's important to remember that we're here to review the guides, not you, so try not to worry if you're unsure about things. I need you to find the information in the guides, not use your own knowledge. There's no time limit, so take as long as you need.

[Give information sheet]

Here's the information sheet explaining more about this. Could you read through and let me know if you have any questions.

[Give consent form]

Here's the consent form. Could you read each statement and initial if you are happy with it, then sign and date at the bottom?

[Complete demographics]

I'll start the recording now.

[Start recording.]

This is study IRAS 235214, participant number [ ] and the date is [ ]

**1.1**	**Could you tell me how you were first trained to prepare and administer intravenous medicines?**
	Probe – what "classroom" training did you have?<br>Probe – what "on the job" training did you have?<br>Probe – how was your competence assessed?
**1.2**	**Since you were trained, what has been your experience of preparing and administering intravenous medicines?**
	Probe – in what clinical settings have you administered intravenous medicines and for how long?<br>Probe – what types of intravenous medicines have you administered?
**1.3**	**What written guidance have you used to help you prepare and administer intravenous medicines?**
	Probe – have you ever used the Medusa guide? Was this online or on paper?<br>Probe – have you ever used the UCL Hospital Medicines Administration Guide?<br>Probe – have you ever used the Injectable Drugs Guide?<br>Probe – have you ever used a guide written within your organisation?

Odd participant numbers: start with bathicillin [ ]

Even participant numbers: start with unimycin [ ]

[Give current copy of intravenous administration guide for bathicillin to participant.]

On screen is a copy of the intravenous administration guide for "bathicillin". You can do everything on this guide that you can do on an ordinary website. I need you to answer the next set of questions by finding the information in this guide, not using your own knowledge.

**2.1**	**How is intravenous bathicillin supplied?**
	Indicative answer: all of:<br>i. Vials<br>ii. 200mg<br>iii. Powder for reconstitution<br>*Probe: does the guide say anything else?*<br>*Probe: sorry I didn't catch that, could you repeat your answer*
**2.2**	**Imagine you are <u>reconstituting</u> 200mg of bathicillin. What do you need to use?**
	Indicative answer: both of:<br>i. 19ml<br>ii. Water (for injections) OR sodium chloride 0.9%<br>*Probe: does the guide say anything else?*
**2.3**	**What infusion solutions can be used to <u>dilute</u> reconstituted bathicillin solution?**
	Indicative answer: both of:<br>i. Sodium chloride 0.9%<br>ii. Glucose 5%<br>*Probe: are there any other solutions?*<br>*Probe: sorry, I didn't quite catch that, could you say it again?*
**2.4**	**What should be monitored before starting a patient on bathicillin?**
	Indicative answer: either:<br>i. Electrolyte disturbances<br>ii. Serum potassium, magnesium and calcium<br>iii. Hypokalaemia, hypomagnesemia and hypocalcaemia
**2.5**	**Suppose you are preparing a dose of 600mg of bathicillin. What size infusion bag should be used?**
	Indicative answer:<br>*i.* 100ml, 250ml, 500ml or 1000ml<br>*Probe: how did you decide that (did they account for reconstituted solution volume)?*

**2.6**	**How much sodium does the B-Cil brand of bathicillin contain?**
	Indicative answer:<br>i. 9.5mmol per vial
**2.7**	**Imagine you're making an infusion of 270mg bathicillin. How much reconstituted solution should you add to the infusion bag?**
	Indicative answer:<br>i. 27ml<br>*Score NF if answer 25.6ml*
**2.8**	**Can you give bathicillin to a patient who is allergic to latex?**
	Indicative answer:<br>i. Yes – natural rubber latex is not used as a material
**2.9**	**Imagine you were giving a dose of 420mg of bathicillin to a patient weighing 70kg. What's the shortest time the infusion should last?**
	Indicative answer:<br>i. 2 hours

[Give current copy of intravenous administration guide for unimycin to participant.]

On screen is a copy of the intravenous administration guide for "unimycin". I need you to answer the next set of questions by finding the information in this guide, not using your own knowledge.

**2.10**	**In general, in what different ways can a dose of intravenous unimycin be given and why?**
	Indicative answer: all of:<br>i. Injection or short infusion for a loading dose<br>ii. Continuous infusion for the maintenance dose<br>*Probe: does the guide say anything else?*<br>*Probe: why are there two options?*
**2.11**	**Suppose you were programming an infusion pump for a continuous infusion of unimycin at a rate of 700mcg/kg/hour for a patient weighing 65kg. What infusion rate do you require in ml per hour?**
	Indicative answer:<br>i. 45.5 ml/hour
**2.12**	**What should you do if a patient develops low blood pressure whilst being given unimycin?**
	Indicative answer: both of:<br>i. Slow the rate of administration, or<br>ii. Stop the infusion<br>*Probe: are there any other options?*
**2.13**	**Can you give an infusion of ciprofloxacin at the same time as an infusion of unimycin through the same cannula?**
	Indicative answer:<br>i. No (they are incompatible)<br>*Probe: what the implication of a drug being incompatible?*
**2.14**	**Suppose you prepare a continuous infusion of unimycin at 1pm on Monday. What is the latest time you must stop using this infusion?**
	Indicative answer:<br>i. 1pm on Tuesday

**2.15**	**Why is extravasation of unimycin likely to be harmful?**
	Indicative answer:<br>i. It has an extreme pH<br>*Probe: what is that pH?*
**2.16**	**Why is it important to <u>double</u> check the correct dose of unimycin has been prescribed?**
	Indicative answer:<br>i. It may be administered as a loading dose followed by a smaller maintenance dose
**2.17**	**What must you do if you need to give undiluted unimycin injection?**
	Indicative answer: Either:<br>i. Give by a central venous access device<br>ii. Give by a central line

If internet access available, show participant electronic calculator on aminophylline monograph:

**2.18**	**Please have a look at this electronic rate calculator. What do you think about how useful this would be in practice?**
	Probe – have you used a calculator like this before?<br>Probe – do you think you would use it?<br>Probe – what benefits do you think this calculator would have?<br>Probe – what disadvantages might this calculator have?<br>Probe – would you trust the calculator?

Thank you for answering those questions. For the rest of the interview, I will ask you some general question about how you found the intravenous administration guides.

**3.1**	**In your opinion, how useful are these guides for supporting the administration of intravenous bathicillin and unimycin?**
	Probe – what makes you say that?<br>Probe – how clear is it that this guide is meant to support the administration of intravenous bathicillin and unimycin?<br>Probe – what aspects of the guides make them useful?<br>Probe – what aspects of the guides make them less useful?
**3.2**	**What do you think of the information included in the guides?**
	Probe – what information is missing?<br>Probe – what information is not necessary?<br>Probe – do you ever use the yellow "i" buttons to find more information?
**3.3**	**What you think of the layout of the guides?**
	Probe – what information did you find it difficult to locate?<br>Probe – what information did you find it easy to locate?
**3.4**	**What do you think of the order of the information in the guides?**
	Probe – which sections of the guides appear in a logical order?<br>Probe – which sections of the guides do not appear in a logical order?
**3.5**	**What do you think of the headings used in the guides?**
	Probe – what headings would you suggest adding and why?<br>Probe – what headings would you suggest taking away and why?<br>Probe – what headings would you suggest changing and why?
**3.6**	**What do you think of the typeface used for the guides?**
	Probe – what do you think of its size?<br>Probe – what do you think of the font?<br>Probe – what do you think of the use of bold, underlined, italic and coloured text?
**3.7**	**What do you think of how understandable the guides are?**
	Probe – which words and sentences did you not understand?<br>Probe – which sections were particularly well explained?<br>Probe – what do you think of the example calculation and infusion rate table
**3.8**	**How satisfying are the guides to use?**
	Probe – what aspects of the guides are easy to use?<br>Probe – what aspects of the guides are hard to use?
**3.9**	**How trustworthy do you think the guides are is?**

	Probe – what aspects of the guide make it more trustworthy?<br>Probe – what aspects of the guide make it less trustworthy?
**3.10**	**Imagine you didn't give bathicillin and unimycin to patients every day, but you had to do it sometimes. Would you want to use these guides to help you?**
	Probe – what aspects of the guides would make you want to use it more or feel good about using it?<br>Probe – what aspects of the guides would make you want to use it less or feel bad about using it?
**3.11**	**What do you think of whether these guides are intended to be used by a nurse doing a job like yours?**
	Probe – what aspects of the guides make them appear more intended for a nurse like you?<br>Probe – what aspects of the guides make them appear less intended for a nurse like you?
**3.12**	**Is there anything else which would make it easier to use the guides?**
	Probe – what makes you say that?
**3.13**	**Those are all the questions that I have. Is there anything else about the guides which you would like to say?**

[Follow up any questions where the participant did not find the answer or did not understand it.]

- Repeat question
- Show them the information
- What made it difficult to find this information?
- What would have made it easier to find or understand this information?

[Stop recorder]

Thank you once again for your time today, this has been a really helpful conversation.

Interview schedule – researcher (rounds 2 and 3)

Effects of User Testing Injectable Medicines Guidance – Phase 1

Thank you for taking the time to speak to me today. Your help with this study is greatly appreciated.

This interview should last about 45-60 minutes, although if you would like to pause at any point, please let me know. If you decide that you no longer want to take part, please also let me know, and we can stop the interview.

The interview will have three parts. In the first part, I will ask you a few questions about your experience of preparing and administering intravenous medicines. Then I will give you two intravenous administration guides for invented medicine called "bathicillin" and "unimycin". I will ask you questions about these medicines and ask you to answer them by finding the information in the guides. In the final part of the interview, I will ask you some questions about how you found using the guide.

It's important to remember that we're here to review the guides, not you, so try not to worry if you're unsure about things. I need you to find the information in the guides, not use your own knowledge. There's no time limit, so take as long as you need.

[Give information sheet]

Here's the information sheet explaining more about this. Could you read through and let me know if you have any questions.

[Give consent form]

Here's the consent form. Could you read each statement and initial if you are happy with it, then sign and date at the bottom?

[Complete demographics]

I'll start the recording now.

[Start recording.]

This is study IRAS 235214, participant number ☐ and the date is ☐

**1.1**	**Could you tell me how you were first trained to prepare and administer intravenous medicines?**
	Probe – what "classroom" training did you have?<br>Probe – what "on the job" training did you have?<br>Probe – how was your competence assessed?
**1.2**	**Since you were trained, what has been your experience of preparing and administering intravenous medicines?**
	Probe – in what clinical settings have you administered intravenous medicines and for how long?<br>Probe – what types of intravenous medicines have you administered?
**1.3**	**What written guidance have you used to help you prepare and administer intravenous medicines?**
	Probe – have you ever used the Medusa guide? Was this online or on paper?<br>Probe – have you ever used the UCL Hospital Medicines Administration Guide?<br>Probe – have you ever used the Injectable Drugs Guide?<br>Probe – have you ever used a guide written within your organisation?

Odd participant numbers: start with bathicillin ☐

Even participant numbers: start with unimycin ☐

[Give current copy of intravenous administration guide for bathicillin to participant.]

On screen is a copy of the intravenous administration guide for "bathicillin". You can do everything on this guide that you can do on an ordinary website. I need you to answer the next set of questions by finding the information in this guide, not using your own knowledge.

**2.1**	**How is intravenous bathicillin supplied?**
	Indicative answer: all of:<br>iv. Vials<br>v. 200mg<br>vi. Powder for reconstitution<br>*Probe: does the guide say anything else?*<br>*Probe: sorry I didn't catch that, could you repeat your answer*
**2.2**	**Imagine you are <u>reconstituting</u> 200mg of bathicillin. What do you need to use?**
	Indicative answer: both of:<br>iii. 19ml<br>iv. Water (for injections) OR sodium chloride 0.9%<br>*Probe: does the guide say anything else?*
**2.3**	**What infusion solutions can be used to <u>dilute</u> reconstituted bathicillin solution?**
	Indicative answer: both of:<br>iii. Sodium chloride 0.9%<br>iv. Glucose 5%<br>*Probe: are there any other solutions?*<br>*Probe: sorry, I didn't quite catch that, could you say it again?*
**2.4**	**What should be monitored before starting a patient on bathicillin?**
	Indicative answer: either:<br>iv. Electrolyte disturbances<br>v. Serum potassium, magnesium and calcium<br>vi. Hypokalaemia, hypomagnesemia and hypocalcaemia
**2.5**	**Suppose you are preparing a dose of 600mg of bathicillin. What size infusion bag should be used?**
	Indicative answer:<br>*ii.* 100ml, 250ml, 500ml or 1000ml<br>*Probe: how did you decide that (did they account for reconstituted solution volume)?*

**2.6**	**How much sodium does the B-Cil brand of bathicillin contain?**
	Indicative answer:<br>ii. 9.5mmol per vial
**2.7**	**Imagine you're making an infusion of 270mg bathicillin. How much reconstituted solution should you add to the infusion bag?**
	Indicative answer:<br>ii. 27ml<br>*Score NF if answer 25.6ml*
**2.8**	**Can you give bathicillin to a patient who is allergic to latex?**
	Indicative answer:<br>ii. Yes – natural rubber latex is not used as a material
**2.9**	**Imagine you were giving a dose of 420mg of bathicillin to a patient weighing 70kg. What's the shortest time the infusion should last?**
	Indicative answer:<br>ii. 2 hours

[Give current copy of intravenous administration guide for unimycin to participant.]

On screen is a copy of the intravenous administration guide for "unimycin". I need you to answer the next set of questions by finding the information in this guide, not using your own knowledge.

**2.10**	**In general, in what different ways can a dose of intravenous unimycin be given and why?**
	Indicative answer: all of:<br>iii. Injection or short infusion for a loading dose<br>iv. Continuous infusion for the maintenance dose<br>*Probe: does the guide say anything else?*<br>*Probe: why are there two options?*
**2.11**	**Suppose you were programming an infusion pump for a continuous infusion of unimycin at a rate of 45.5 mg/hour. What infusion rate do you require in ml per hour?**
	Indicative answer:<br>ii. 45.5 ml/hour
**2.12**	**What should you do if a patient develops low blood pressure whilst being given unimycin?**
	Indicative answer: both of:<br>iii. Slow the rate of administration, or<br>iv. Stop the infusion<br>*Probe: are there any other options?*
**2.13**	**Can you give an infusion of ciprofloxacin at the same time as an infusion of unimycin through the same cannula?**
	Indicative answer:<br>ii. No (they are incompatible)<br>*Probe: what the implication of a drug being incompatible?*
**2.14**	**Suppose you prepare a continuous infusion of unimycin at 1pm on Monday. What is the latest time you must stop using this infusion?**
	Indicative answer:<br>ii. 1pm on Tuesday

**2.15**	**Why is extravasation of unimycin likely to be harmful?**
	Indicative answer:<br>ii. It has an extreme pH<br>*Probe: what is that pH?*
**2.16**	**Why is it important to <u>double</u> check the correct dose of unimycin has been prescribed?**
	Indicative answer:<br>ii. It may be administered as a loading dose followed by a smaller maintenance dose
**2.17**	**What must you do if you need to give undiluted unimycin injection?**
	Indicative answer: Either:<br>iii. Give by a central venous access device<br>iv. Give by a central line

If internet access available, show participant electronic calculator on aminophylline monograph:

**2.18**	**Please have a look at this electronic rate calculator. What do you think about how useful this would be in practice?**
	Probe – have you used a calculator like this before?<br>Probe – do you think you would use it?<br>Probe – what benefits do you think this calculator would have?<br>Probe – what disadvantages might this calculator have?<br>Probe – would you trust the calculator?

Thank you for answering those questions. For the rest of the interview, I will ask you some general question about how you found the intravenous administration guides.

**3.1**	**In your opinion, how useful are these guides for supporting the administration of intravenous bathicillin and unimycin?**
	Probe – what makes you say that?<br>Probe – how clear is it that this guide is meant to support the administration of intravenous bathicillin and unimycin?<br>Probe – what aspects of the guides make them useful?<br>Probe – what aspects of the guides make them less useful?
**3.2**	**What do you think of the information included in the guides?**
	Probe – what information is missing?<br>Probe – what information is not necessary?<br>Probe – do you ever use the yellow "i" buttons to find more information?
**3.3**	**What you think of the layout of the guides?**
	Probe – what information did you find it difficult to locate?<br>Probe – what information did you find it easy to locate?
**3.4**	**What do you think of the order of the information in the guides?**
	Probe – which sections of the guides appear in a logical order?<br>Probe – which sections of the guides do not appear in a logical order?
**3.5**	**What do you think of the headings used in the guides?**
	Probe – what headings would you suggest adding and why?<br>Probe – what headings would you suggest taking away and why?<br>Probe – what headings would you suggest changing and why?
**3.6**	**What do you think of the typeface used for the guides?**
	Probe – what do you think of its size?<br>Probe – what do you think of the font?<br>Probe – what do you think of the use of bold, underlined, italic and coloured text?
**3.7**	**What do you think of how understandable the guides are?**
	Probe – which words and sentences did you not understand?<br>Probe – which sections were particularly well explained?<br>Probe – what do you think of the example calculation and infusion rate table
**3.8**	**How satisfying are the guides to use?**
	Probe – what aspects of the guides are easy to use?<br>Probe – what aspects of the guides are hard to use?
**3.9**	**How trustworthy do you think the guides are is?**

	Probe – what aspects of the guide make it more trustworthy?<br>Probe – what aspects of the guide make it less trustworthy?
**3.10**	**Imagine you didn't give bathicillin and unimycin to patients every day, but you had to do it sometimes. Would you want to use these guides to help you?**
	Probe – what aspects of the guides would make you want to use it more or feel good about using it?<br>Probe – what aspects of the guides would make you want to use it less or feel bad about using it?
**3.11**	**What do you think of whether these guides are intended to be used by a nurse doing a job like yours?**
	Probe – what aspects of the guides make them appear more intended for a nurse like you?<br>Probe – what aspects of the guides make them appear less intended for a nurse like you?
**3.12**	**Is there anything else which would make it easier to use the guides?**
	Probe – what makes you say that?
**3.13**	**Those are all the questions that I have. Is there anything else about the guides which you would like to say?**

[Follow up any questions where the participant did not find the answer or did not understand it.]

- Repeat question
- Show them the information
- What made it difficult to find this information?
- What would have made it easier to find or understand this information?

[Stop recorder]

Thank you once again for your time today, this has been a really helpful conversation.
