# Supplementary material for: User Testing to Improve Retrieval and Comprehension of Information in Guidelines to Improve Medicines Safety
Source: J Patient Saf. 2020 Jun 22;18(1):e172–9. doi: 10.1097/PTS.0000000000000723 (PMC7612138; doi:10.1097/PTS.0000000000000723)
Supplement: SUPPLEMENTARY MATERIAL [file pts-18-e172-s003.pdf]

User testing to improve retrieval and comprehension of information in guidelines to improve medicines safety

Supplementary File 3

IMG guides tested in round 2

(changes and the reasons for them are annotated)

NHS

Injectable Medicines Guide

Bathicillin (B-Cil®) Date published: 18th June 2018

Intravenous - ADULT

Click on the ⓘ icons for background information

Before treatment [1]

Check patient's serum potassium, magnesium and calcium levels. If they are low, they should be corrected. Discuss with the prescriber.

Summary – preparation and administration

Only give by IV infusion after reconstitution and further dilution:

1. Add 19 mL of water for injections or sodium chloride 0.9% to each vial
2. **This solution contains 10 mg bathicillin in 1 mL**
3. Further dilute the reconstituted solution:
   - **If bathicillin dose 120-500 mg:** add the required volume of reconstituted solution to 50 mL or 100 mL of sodium chloride 0.9% or glucose 5%
   - **If bathicillin dose 501-1000 mg:** add the required volume of reconstituted solution to 100 mL or 250 mL of sodium chloride 0.9% or glucose 5%
4. Give over 1 to 3 hours via an infusion pump. Calculate the shortest length of the infusion using the following equation:

$$\text{Shortest length of infusion (hours)} = \frac{\text{Dose (mg)}}{3 \times \text{Patient weight (kg)}}$$

Part 1: Preparation and administration guidance

(See part 2 for other detailed information)

Presentation of medicine [1][5]

Vials containing 200 mg bathicillin powder for reconstitution

Method of administration [1] ⓘ

Only give by IV infusion after reconstitution and further dilution

Preparation [1] ⓘ

1. Add 19 mL of water for injections or sodium chloride 0.9% to each vial:
   - Gently swirl to ensure the powder has completely dissolved and no particles are visible
   - **Do not shake**
   - Discard if water for injections/sodium chloride 0.9% is not pulled into the vial by the vacuum

2. **This reconstituted solution contains 10 mg bathicillin in 1 mL**

3. Further dilute the reconstituted solution:
   - **If bathicillin dose 120-500 mg:** add the required volume of reconstituted solution to 50 mL or 100 mL of sodium chloride 0.9% or glucose 5%
   - **If bathicillin dose 501-1000 mg:** add the required volume of reconstituted solution to 100 mL or 250 mL of sodium chloride 0.9% or glucose 5%

   - If these dilutions are not suitable, dilute the required volume of reconstituted solution to give a final concentration of 0.5-5 mg bathicillin in 1 mL
   - Remember to add the volume of reconstituted solution to the volume of diluent when doing this calculation

Administration [1] ⓘ

- Flush with sodium chloride 0.9% or glucose 5%

- Give over 1 to 3 hours via an infusion pump
- Calculate the shortest length of the infusion using the following equation:

$$\text{Shortest length of infusion (hours)} = \frac{\text{Dose (mg)}}{3 \times \text{Patient weight (kg)}}$$

- **Click on this link for a table** showing the maximum bathicillin dose which can be infused in different times for patients of different weights

Adverse effects and monitoring [1] ⓘ

These adverse effects may occur during or shortly after IV administration:

- Flushing and nausea – if severe, consider stopping treatment
- Anaphylaxis-like reactions – including fever, sweating, tachycardia, chest tightness, dyspnoea, faintness, pruritus, rash
- Visual disturbances
- Peripheral oedema
- Pyrexia
- Headache
- Abdominal pain
- Vomiting

Extravasation [9] ⓘ

Manufacturer has no information

Compatibility [1] ⓘ

Do not infuse with any other medicines

The reconstituted solution can be diluted with:

- Sodium chloride 0.9%
- Glucose 5%

- Glucose 5% and lactated Ringer's solution
- Glucose 5% in sodium chloride 0.45%
- Glucose 5% in sodium chloride 0.9%
- Hartmann's solution (compound sodium lactate)
- 20 mmol potassium chloride in glucose 5%
- Sodium chloride 0.45%

Part 2: Other information

Other comments [1] ⓘ

Commented [MJ1]: Users suggested adding "official branding", to increase credibility.

Commented [MJ2]: Drug name moved from top right to top left. Repeated names deleted from next line – users pointed out name repeated 2-3 times at top of document.

Commented [MJ3]: Date moved to top at user suggestion. Users suggested the date of publication added credibility, but was easily missed at the bottom.

Commented [MJ4]: Route and "Adult" moved under drug name, so more easily seen compared with being on the right.

Commented [MJ5]: Suggested by users, who almost all assumed the icon was part of the title and did not realise a click provided more information.

Commented [MJ6]: Format of titles changed to bold white on dark blue block to better differentiate sections, even when printed in B&W (as many users said they do, even if they shouldn't).

Commented [MJ7]: Users found it difficult to locate this information in "Other comments" section, so new section created.

Commented [MJ8]: Summary section added, as requested by users, who often stated that a lot of scrolling and reading was required and that time was limited.

Commented [MJ9]: Bullet points added (here and elsewhere).

Commented [MJ10]: Bold to emphasise key point missed during user testing (here and elsewhere).

Commented [MJ11]: Suggested dilutions given, rather than requiring user to do the calculation. This was a common cause of error among users of the original guide.

Commented [MJ12]: Some users could not calculate length of infusion from "3mg/kg/hour". Users found example calculations useful, and requested more of them.

Commented [MJ13]: Users valued referencing, as it added credibility, but it did occasionally lead to difficulty in reading the text. Therefore, referencing moved to the heading of each section.

Commented [MJ14]: Reconstitution and dilution section combined into "preparation". Users did not always understand the distinction between reconstitution and dilution. This will also make it clearer how to prepare a medicine in situations where different doses or rates of administration require different instructions, as can have one named comprehensive list of directions for each scenario.

Commented [MJ15]: Sentence case to improve readability. Bold for emphasis.

Commented [MJ16]: Bold text and spacing to emphasise this point which was missed by most users.

Commented [MJ17]: Suggested dilutions given, rather than requiring user to do the calculation. This was a common cause of error among users of the original guide.

Commented [MJ18]: 500 mcg removed – made reading the concentration more confusing and this is not a prescribing situation where a 1000x dose error is possible. Changed from "0.5 mg to 5 mg" to "0.5-5mg" to reduce number of words, emphasise that a range of concentration are available and emphasise that 0.5mg is half a mg, not 5mg.

Commented [MJ19]: Users often forgot to do this – can be significant with bathicillin.

Commented [MJ20]: Users commented that there was a lot of scrolling. This was partly because administration rate information was given first, but this is not needed until after dilution.
So, new section on administration created. Incorporates rate information from "Method of administration" and "Flushing" information".

Commented [MJ21]: Some users could not calculate length of infusion from "3mg/kg/hour". Users found example calculations useful, and requested more of them.

Commented [MJ22]: Users also found example tables helpful, but often did not notice the link. Text in bold to help this, but also blue underlined, as this is a common web convention for links.

Commented [MJ23]: Title shortened as per user suggestion.

Commented [MJ24]: Description of what type of adverse effects are included moved here from the title.

Commented [MJ25]: Information on how common these adverse effects are is not necessary – removed.

Commented [MJ26]: Brackets replaced by a dash.

Commented [MJ27]: Heading shortened as suggested by user ("it's all useful in clinical practice, isn't it?")

Commented [MJ28]: List put into alphabetical order, except most common solutions first which are followed by a small gap.

Commented [MJ29]: Put common name first rather than in brackets.

- Bathicillin may cause QT interval prolongation
- In patients with eGFR <50mL/min, a cyclodextrin excipient can accumulate. Give these patients oral bathicillin unless a risk benefit assessment justifies the use of IV route.
- Carefully monitor patients receiving bathicillin for hepatic toxicity

Special handling precautions [10]

Do not handle bathicillin if pregnant, planning pregnancy or breastfeeding. Teratogenic in rats.

Latex content [9a-c]

- **B-Cil (Pfizer):** Natural rubber latex is not used as a material in the manufacture of this product or in the container or packaging. October 2015
- **Teva:** Natural rubber latex is not used as a material in the manufacture of this product or in the container or packaging. September 2016
- **Panpharma:** Natural rubber latex is not used as a material in the manufacture of this product or in the container or packaging. August 2016

Sodium content [9a-c]

- **B-Cil (Pfizer):** 9.5 mmol per vial
- **Teva and Panpharma:** free from sodium

pH [9a-c]

- **B-Cil (Pfizer):** undiluted solution pH 5.5 to 7.5
- **Teva:** pH 6.1
- **Panpharma:** no information

Osmolarity / osmolality [9a-c]

- **B-Cil (Pfizer):** reconstituted solution is approximately 507 mOsmol/L
- **Teva:** 210 mOsm/kg
- **Panpharma:** no information

Infusion pump to use for the infusion therapy category [7]

- Infusion is 'Therapy Category' B
- The infusion pump used should have critical performance parameters described for 'therapy category' B or higher

Product risk factors

Risk assessment of a common preparation (as required by NPSA Patient Safety Alert 20)

Risk factors for bathicillin infusion 0.5-5mg/mL: Therapeutic risk; Use of concentrate; Complex calculation; Complex preparation; Reconstitute vial; Use of multiple and part container; Use of infusion pump.

1	2	3	4	5	6	7		**TOTAL RISK FACTORS:** 7	**OVERALL RISK RATING:** Red

Current suppliers

Actavis UK Limited
This product was not available when the monograph was prepared
SPC for Bathicillin Actavis 200mg Powder for Solution for Infusion
PIL for Bathicillin 200mg Powder for Solution for Infusion

Panpharma UK Ltd
Supplies Xellia Pharmaceuticals product
SPC for Bathicillin 200mg Powder for Solution for Infusion
PIL for Bathicillin 200mg Powder for Solution for Infusion

Pfizer Limited
Trade names: B-CIL
SPC for B-CIL 50 mg and 200 mg film-coated tablets, B-CIL 200 mg powder for solution for infusion,B-CIL 40 mg/ml powder for oral suspension
SPC for Bathicillin 200mg Powder Solution Infusion
PIL for B-CIL 200mg powder for solution for infusion
PIL for B-CIL 200 mg powder and solvent for solution for infusion
PIL for Bathicillin 200mg Powder Solution Infusion

Teva UK Limited
PIL Bathicillin Teva 200mg powder for solution for infusion
SPC Bathicillin Teva 200 mg Powder for Solution for Infusion

References

1. Summary of Product Characteristics
   a) B-Cil®, Pfizer. Last updated 06/2016
   b) Bathicillin, Panpharma. Last updated 06/2015
   c) Bathicillin, Pfizer. Last updated 03/2014
   d) Bathicillin, Teva UK Ltd. Last updated 25/02/2016
2. Martindale "The Complete Drug Reference" accessed via www.thomsonhc.com on 30/08/2013
3. American Hospital Formulary Service Drug Information 2011
4. ASHP 'Handbook on Injectable Drugs' 17th Edition pg 1150
5. British National Formulary No. 70, September 2015 pg 521 and 522
6. British National Formulary for Children 2015-2016 pg 342
   a) Evelina London Paediatric Formulary
7. MHRA guidance for healthcare professions on using and managing infusion systems
   a) Specimen High Risk Injectable Medicines List – November 2016
8. Development of the UK Vessel Health and Preservation (VHP) framework: a multi-organisational collaborative; 2016
9. a) Drug company name: Pfizer. Date contacted: October 2015
   b) Drug company name: Teva. Date contacted: September 2016
   c) Drug company name: Panpharma (PL holder Xellia). Date contacted: August 2016
10. Pfizer Material Safety Data Sheet 2007

Version MDJ2

Commented [MJ30]: Numbered list replaced by bullet points, as list is non-sequential and points are of equal importance.

Commented [MJ31]: Passive voice switched to active voice (here and elsewhere).

Commented [MJ32]: Title shortened (units specified below).

Commented [MJ33]: Osmolarity moved to the end of the four "content" sections, as it is the least used.

Maximum bathicillin dose which can be infused in 1 hour, 80 minutes and 2 hours for patients of different weights:

**Weight (kg)**	**Infusion time**		
	**1 hour**	**80 minutes**	**2 hours**
	Maximum bathicillin dose which can be given in infusion time		
40	120 mg	160 mg	240 mg
50	150 mg	200 mg	300 mg
60	180 mg	240 mg	360 mg
70	210 mg	280 mg	420 mg
80	240 mg	320 mg	480 mg
90	270 mg	360 mg	540 mg
100	300 mg	400 mg	600 mg
110	330 mg	440 mg	660 mg
120	360 mg	480 mg	720 mg
130	390 mg	520 mg	780 mg
140	420 mg	560 mg	840 mg

Unimycin Date published: 18th June 2018

Intravenous - ADULT

Click on the icons for background information

Before treatment

Safety alert: double check the correct dose has been prescribed, because unimycin may be administered as a loading dose followed by a smaller maintenance dose (see 'Other Comments' section in Part 2 below).

Commented [MJ34]: Bold "Safety alert" added to emphasise importance of this warning as many users thought this unnecessary as all doses should be checked.

Commented [MJ35]: Users found it difficult to locate this information in the "method of administration" section, so relocated here. Sentence reordered, to state the action first ("double check…") followed by explanation – to make it easier to locate key information. In addition, "because" added to make it clear the second part of the sentence is an explanation, as many users were not sure why double checking was required.

Summary – preparation and administration

IV injection or **short IV infusion** – for loading doses:

- Dilute dose to 100 mL with sodium chloride 0.9% or glucose 5%
- Give slowly over at least 20 minutes, using an infusion pump
- Do **not** give faster than 25 mg per minute

OR

Continuous IV infusion – for maintenance doses:

- Dilute to a concentration of 1 mg in 1 mL with sodium chloride 0.9% or glucose 5%
- Administer at prescribed rate using an infusion pump

Part 1: Preparation and administration guidance

(See part 2 for other detailed information)

Commented [MJ36]: Highlighting there is another section.

Presentation of medicine [1]

Glass ampoules containing unimycin. Strength: 250 mg in 10 mL

Method of administration [1][5]

- **IV injection** or **short IV infusion** – for loading doses
- **Continuous IV infusion** – for maintenance doses

Commented [MJ37]: Some users did not understand why there were different methods of administration, so have reordered information to present method first, follow by reason, using the word "for" to imply an explanation.

Preparation [1]

IV injection or **short IV infusion** – for loading doses:

- **Dilute dose to 100 mL with sodium chloride 0.9% or glucose 5%**
  1. Remove volume equivalent to the required dose from a 100 mL bag of sodium chloride 0.9% or glucose 5%
  2. Add required dose of unimycin injection to the bag

Commented [MJ38]: More detail of preparation given. Users often described confusion over whether or not to remove a volume from an infusion bag. They also valued specific advice in all situations.

- If patient is fluid restricted, you can give higher concentrations (or undiluted unimycin) by a central venous access device

Commented [MJ39]: Reordered to give situation where this is acceptable first, so most people don't have to read on.

OR

Continuous IV infusion – for maintenance doses:

- **Dilute to a concentration of 1 mg in 1 mL with sodium chloride 0.9% or glucose 5%**
- For example, to make a 500 mg in 500 mL solution:
  1. Remove 20 mL from a 500 mL bag of sodium chloride 0.9% or glucose 5%
  2. Add 500 mg (20 mL) of unimycin injection to the bag

- If patient is fluid restricted, you can give higher concentrations (or undiluted unimycin) by a central venous access device

Expiry time to write on label of continuous infusion [4]

24 hours

Administration [1][5]

- Flush with sodium chloride 0.9% or glucose 5%

IV injection or **short IV infusion** – for loading doses:

- Give slowly over at least 20 minutes, using an infusion pump
- Do **not** give faster than 25 mg per minute
- Give higher concentrations (or undiluted unimycin) via a central venous access device

OR

Continuous IV infusion – for maintenance doses:

- Administer at prescribed rate using an infusion pump
- Give higher concentrations (or undiluted unimycin) via a central venous access device
- If necessary, calculate the infusion rate using the following equation:

Commented [MJ40]: Example calculations moved into administration section, as some users missed them in a separate section. Calculation changed to be one needed by staff administering (rather than prescribing) unimycin.

$$\text{Unimycin infusion rate (mL/hour)} = \frac{\text{Prescribed rate (mg/hour)}}{\text{Infusion concentration (mg/mL)}}$$

- For example, for a prescribed rate of **35 mg/hour** using an infusion concentration of **1 mg in 1 mL**, the calculation is as follows:

$$\text{Unimycin infusion rate} = \frac{\mathbf{35}\ \text{(mg/hour)}}{\mathbf{1}\ \text{(mg/mL)}} = 35\ \text{ml/hour}$$

- **Prescribers:** To calculate the infusion rate using the patient's body weight, see 'Example dose calculation' section in 'Part 2' below

Commented [MJ41]: Rate calculation for prescribers in a separate section, signposted from here.

Adverse effects and monitoring [1][5]

These adverse effects may occur during or shortly after IV administration:

- Hypotension, arrhythmias and convulsions – especially if given rapidly
- Hypersensitivity reactions
- Nausea and vomiting
- Dizziness, headache, CNS stimulation and insomnia

Commented [MJ42]: Bullet points added and effects divided by organ system or those that occur if given rapidly.

If acute adverse effects occur, slow the rate or stop the infusion for 5-10 minutes

Commented [MJ43]: Users had difficulty finding this advice when it was in the "methods" section. They expected it to be in "adverse effects", so moved it there and highlighted with bold.

Monitor:

- ECG, heart rate and blood pressure
- Plasma-unimycin levels according to local policy
- Serum potassium levels if therapy is on-going

Extravasation

Likely to cause tissue damage due to extreme pH (see 'pH' section in 'Part 2' below)

Commented [MJ44]: Wording changed to make it clear that extreme pH is what make tissue damage likely.

Commented [MJ45]: Add signpost to pH section, as users were unsure what pH was. pH information too long to include here.

Compatibility [1][4]

Compatible infusions (it is assumed that medicines meet close to the vascular access device):

- Esmolol
- Fluconazole, foscarnet
- Labetolol, linezolid
- Meropenem, micafungin
- Nicardipine
- Pancuronium, piperacillin-tazobactam, potassium chloride
- Tacrolimus, terbutaline
- Vecuronium

Incompatible drugs:

- Amiodarone
- Ciprofloxacin, clarithromycin, clindamycin
- Dobutamine
- Ondansetron
- Phenytoin sodium
- Salbutamol
- Vancomycin

Compatible infusion solutions:

- Glucose 10%, glucose 20%
- Hartmann's solution (compound sodium lactate)
- Ringers solution for injection
- Sodium chloride/glucose mixtures

Part 2: Other information

Other comments [1][2][5]

- A loading dose is not normally given to adults or children taking oral unimycin; if considered necessary, delay treatment until a serum unimycin level is available
- Store ampoules at room temperature (25ºC or below) and in original packaging to protect from light.
- Discard if the ampoule contents are discoloured
- Recommended dilutions are based on common practice in the UK

Commented [MJ46]: Numbering changed to bullets, as not sequential.

Example dose calculation

Continuous IV infusion – for maintenance doses:

- The initial maintenance infusion rate should be less than 500-700 micrograms/kg/hour (or 300 micrograms/kg/hour in older patients)
- Use ideal body weight in obese patients
- Calculate the infusion rate using the following equation:

$$\text{Unimycin infusion rate (mL/hour)} = \frac{\text{Dose (micrograms/kg/hour)} \times \text{Patient weight (kg)}}{1{,}000 \times \text{Infusion concentration (mg/mL)}}$$

- **Example:** For a **70 kg** patient on a maintenance dose of **500 micrograms/kg/hour** using a solution of **1 mg in 1 mL**, the calculation is as follows:

$$\text{Unimycin infusion rate} = \frac{\mathbf{500}\text{ (micrograms/kg/hour)} \times \mathbf{70}\text{ (kg)}}{1{,}000 \times \mathbf{1}\text{ (mg/mL)}} = 35\text{ mL/hour}$$

- [Click on this link for a table] showing unimycin infusion rates for selected patient weights (adults)
- The prescriber should adjust the rate and duration of the infusion according to plasma-unimycin levels and patient requirements

Commented [MJ47]: Users also found example tables helpful, but often did not notice the link. Text in bold to help this, but also blue underlined, as this is a common web convention for links.

Commented [MJ48]: Making it clear who's responsibility this is, as users explained that sometimes the original guide didn't seem to "understand" what their role was (i.e. to administer a prescribed dose, not make dose adjustments).

Commented [MJ49]: Prescribing guidance moved from "Method of Administration" section, as more relevant to prescribers than people giving the dose.

Latex content [9a-b]

These products are **not** made with natural rubber latex, and have **not** been in contact with natural rubber latex during manufacture (Jul 15).

Sodium content [1]

Negligible (undiluted)

pH [4][9b][12]

- Undiluted: pH 8.6 to 10
- Diluted to 1 mg in 1 mL in sodium chloride 0.9% or glucose 5%: pH 9 to 9.2

Osmolarity / osmolality [4]

- Undiluted: 170 mOsmol/L
- 250 mg unimycin diluted in 100 mL sodium chloride 0.9% or glucose 5%: 290-320 mOsmol/L

Commented [MJ50]: Description of solution moved before osmolarity.

Infusion pump to use for the infusion therapy category [7]

- Infusion is 'Therapy Category' A due to narrow therapeutic margin
- The infusion pump used should have critical performance parameters described for 'therapy category' A

Product risk factors

Risk assessment of common preparations (as required by NPSA Patient Safety Alert 20):

Loading dose (adult): A risk assessment carried out on a dose of 300 mg unimycin in 100 mL sodium chloride 0.9% prepared in a clinical area identified the following risk factors: Therapeutic risk; Use of a concentrate; Use of part vial or more than one vial; Use of pump.

- Usual total risk factors: 4
- Overall risk rating: amber

Maintenance infusion (adult): A risk assessment carried out on concentration of unimycin 1 mg in 1 mL (500 mg in 500 mL sodium chloride 0.9%) prepared in a clinical area identified the following risk factors: Therapeutic risk; Use of a concentrate; Complex calculation; Use of part vial or more than one vial; Use of pump.

- Usual total risk factors: 5
- Overall risk rating: amber

Current suppliers

Concordia International - formerly AMCo
Supplies Mercury Pharma product
SPC for Unimycin hydrate 25mg/ml Solution for injection
PIL for Unimycin hydrate 25mg/ml Solution for injection

hameln pharmaceuticals limited
SPC for Unimycin Injection BP (Hameln)
PIL for Unimycin Injection BP (Hameln)

References

11. Summary of Product Characteristics
12. a) Mercury Pharma (Supplier Amidipharm Mercury); Unimycin hydrate 25mg/mL. Last revised 23/08/2012.
13. b) Hameln. Unimycin injection. Last revised 23/01/2015.
14. Martindale 'The Complete Drug Reference' accessed via MedicinesComplete on 27/05/2015
15. American Hospital Formulary Service 'Drug Information' accessed via MedicinesComplete on 27/05/2015
16. ASHP 'Handbook on Injectable Drugs' accessed via MedicinesComplete on 27/05/2015
17. British National Formulary Online accessed on 270/5/2015
18. British National Formulary for Children Online accessed on 27/05/2015
19. MHRA guidance for healthcare professions on using and managing infusion systems
20. a) Specimen High Risk Injectable Medicines List – November 2016
21. Development of the UK Vessel Health and Preservation (VHP) framework: a multi-organisational collaborative; 2016
22. a) Drug company name: Amdipharm Mercury. Date of contact: 07/07/2015
23. b) Drug company name: hameln Pharmaceuticals. Date of contact: 06/07/2015
24. Guy's and St. Thomas', King's College and University Lewisham Hospitals Paediatric Formulary 9th Edition accessed at www.guysandstthomas.nhs.uk/resources/publications/formulary/paediatric-formulary-9th-edition.pdf on 27/05/2015
25. NHS Lothian. Critical Care Guidelines. Unimycin. December 2012. Accessed on 27/05/2015 at www.nhslothian.scot.nhs.uk/Services/A-Z/CriticalCare/DrugsList/DrugsList/unimycin.pdf
26. Quality Assurance Department, Charing Cross Hospital July11

Version MDJ2

ADULT: Unimycin infusion rate table for selected weights

Maintenance infusion (using a solution diluted to 1 mg in 1 mL)

**Weight Kg**	**Maintenance dose**		
	**500 micrograms/kg/hour**	**600 micrograms/kg/hour**	**700 micrograms/kg/hour**
	Unimycin infusion rate (mL/hour) **using a 1 mg in 1 mL solution**		
40	20	24	28
50	25	30	35
60	30	36	42
70	35	42	49
80	40	48	56
90	45	54	63
100	50	60	70
110	55	66	77
120	60	72	84
130	65	78	91
140	70	84	98
