# Supplementary material for: User Testing to Improve Retrieval and Comprehension of Information in Guidelines to Improve Medicines Safety
Source: J Patient Saf. 2020 Jun 22;18(1):e172–9. doi: 10.1097/PTS.0000000000000723 (PMC7612138; doi:10.1097/PTS.0000000000000723)
Supplement: SUPPLEMENTARY MATERIAL [file pts-18-e172-s004.pdf]

User testing to improve retrieval and comprehension of information in guidelines to improve medicines safety

Supplementary File 4

IMG guides tested in round 3

(changes and the reasons for them are annotated)

Bathicillin (B-Cil®)

Intravenous - ADULT

Click on the ⓘ icons for background information

Preparation and Administration Summary

Before treatment (1)

Check patient's serum potassium, magnesium and calcium levels. If they are low, they should be corrected. Discuss with the prescriber

Presentation of medicine (1)(5)

Vials containing 200 mg bathicillin powder for reconstitution

Method of administration (1) ⓘ

Only give by IV infusion using an infusion pump after reconstitution and further dilution

Preparation (1)(10) ⓘ

Do not handle bathicillin if pregnant, planning pregnancy or breastfeeding.

- Add 19 mL of water for injections or sodium chloride 0.9% to each vial. **This makes 20 mL containing 200 mg bathicillin in each vial**
  - Do not shake – gently swirl to ensure the powder has completely dissolved and no particles are visible
  - Discard if water for injections/sodium chloride 0.9% is not pulled into the vial by the vacuum
- Dilute the required dose with sodium chloride 0.9% or glucose 5%:
  - **If bathicillin dose 120-500 mg:** add to 50 mL or 100 mL bag
  - **If bathicillin dose 501-1000 mg:** add to 100 mL or 250 mL bag
  - If these dilutions are not suitable, dilute the required dose to give a final concentration of 0.5-5 mg bathicillin in 1 mL
  - Remember to add the volume of reconstituted solution to the volume of diluent when doing this calculation

Administration (1) ⓘ

- Flush with sodium chloride 0.9% or glucose 5%
- Give by IV infusion using an infusion pump
- Click on this link for a table showing the length of infusion for different doses and patient weights

OR

- Calculate the length of the infusion using the following equation:

$$\text{Length of infusion (hours)} = \frac{\text{Dose (mg)}}{3 \times \text{Patient weight (kg)}}$$

Compatibility (1) ⓘ

Do not infuse with any other medicines

The reconstituted solution can be diluted with:

- Sodium chloride 0.9%
- Glucose 5%
- Glucose 5% and lactated Ringer's solution
- Glucose 5% in sodium chloride 0.45%
- Glucose 5% in sodium chloride 0.9%
- Hartmann's solution (compound sodium lactate)
- 20 mmol potassium chloride in glucose 5%
- Sodium chloride 0.45%

Adverse effects and monitoring (1) ⓘ

These adverse effects may occur during or shortly after IV administration:

- Flushing and nausea – if severe, consider stopping treatment
- Anaphylaxis-like reactions – including fever, sweating, tachycardia, chest tightness, dyspnoea, faintness, pruritus, rash
- Visual disturbances
- Peripheral oedema
- Pyrexia
- Headache
- Abdominal pain
- Vomiting

Extravasation (9) ⓘ

Manufacturer has no information

Detailed Information

Other comments (1) ⓘ

- Bathicillin may cause QT interval prolongation
- In patients with eGFR <50mL/min, a cyclodextrin excipient can accumulate. Give these patients oral bathicillin unless a risk benefit assessment justifies the use of IV route.
- Carefully monitor patients receiving bathicillin for hepatic toxicity

Latex content (9a-c) ⓘ

- **B-Cil (Pfizer):** Natural rubber latex is not used as a material in the manufacture of this product or in the container or packaging. October 2015
- **Teva:** Natural rubber latex is not used as a material in the manufacture of this product or in the container or packaging. September 2016
- **Panpharma:** Natural rubber latex is not used as a material in the manufacture of this product or in the container or packaging. August 2016

Sodium content (9a-c) ⓘ

- **B-Cil (Pfizer):** 9.5 mmol per vial
- **Teva and Panpharma:** free from sodium

Commented [MJ1]: Summary removed. Many nurses liked it, but:
- Advisory group concern.
- 5/9 said they would not read below it.
- 2/9 wanted only the full content.
- Made it harder to see "before Treatment" and "Presentation".
- Some nurses said there were too many headings.

Commented [MJ2]: Colour of most bars made lighter, to highlight preparation and administration sections. Also will make page less "heavy" on the eye.

Commented [MJ3]: Added pump direction at user's request.

Commented [MJ4]: Move from handling precautions section, as unlikely to be read otherwise.

Commented [MJ5]: Concentration moved, as many nurses missed it as point 2. It is a point of info, not an action, so should not be a numbered point anyway. Put in same line as reconstitution instructions, as bullet points may not be read. Units changed to 200mg/20ml (from 10mg/ml), as nurses more often think in units of "one vial" than per unit volume. Line length shortened so not too long and also to place 20ml at start of line, to make more obvious.

Commented [MJ6]: "Do not shake" and "Gently swirl" combined, as linked concepts

Commented [MJ7]: Line length shortened for easier reading.

Commented [MJ8]: Simplified wording based on vancomycin monograph, at nurse's suggestion.

Commented [MJ9]: Removed 1-3 hours, as this conflicts with 3mg/kg/hr and causes confusion.

Commented [MJ10]: Table link put first to encourage use of table and thus avoidance of calculation errors.

Commented [MJ11]: Compatibility moved before ADRs, as nurses say this is a more logical order.

Commented [MJ12]: Extra line space to separate sections.

Commented [MJ13]: Left align title so more easily seen.

Commented [MJ14]: Handling precaution section removed as information added to preparation section.

pH[9a-c]

- **B-Cil (Pfizer):** undiluted solution pH 5.5 to 7.5
- **Teva:** pH 6.1
- **Panpharma:** no information

Osmolarity / osmolality [9a-c]

- **B-Cil (Pfizer):** reconstituted solution is approximately 507 mOsmol/L
- **Teva:** 210 mOsm/kg
- **Panpharma:** no information

Infusion pump performance [7]

Commented [MJ15]: Title made shorter at user's suggestion.

- Infusion is 'Therapy Category' B
- The infusion pump used should have critical performance parameters described for 'therapy category' B or higher

Product risk factors

Risk assessment of a common preparation (as required by NPSA Patient Safety Alert 20)

Risk factors for bathicillin infusion 0.5-5mg/mL: Therapeutic risk; Use of concentrate; Complex calculation; Complex preparation; Reconstitute vial; Use of multiple and part container; Use of infusion pump.

1 2 3 4 5 6 7 **TOTAL RISK FACTORS:** 7 **OVERALL RISK RATING:** Red

Current suppliers

Actavis UK Limited
This product was not available when the monograph was prepared
SPC for Bathicillin Actavis 200mg Powder for Solution for Infusion
PIL for Bathicillin 200mg Powder for Solution for Infusion

Panpharma UK Ltd
Supplies Xellia Pharmaceuticals product
SPC for Bathicillin 200mg Powder for Solution for Infusion
PIL for Bathicillin 200mg Powder for Solution for Infusion

Pfizer Limited
Trade names: B-CIL
SPC for B-CIL 50 mg and 200 mg film-coated tablets, B-CIL 200 mg powder for solution for infusion,B-CIL 40 mg/ml powder for oral suspension
SPC for Bathicillin 200mg Powder Solution Infusion
PIL for B-CIL 200mg powder for solution for infusion
PIL for B-CIL 200 mg powder and solvent for solution for infusion
PIL for Bathicillin 200mg Powder Solution Infusion

Teva UK Limited
PIL Bathicillin Teva 200mg powder for solution for infusion
SPC Bathicillin Teva 200 mg Powder for Solution for Infusion

References

1. Summary of Product Characteristics
   a) B-Cil®, Pfizer. Last updated 06/2016
   b) Bathicillin, Panpharma. Last updated 06/2015
   c) Bathicillin, Pfizer. Last updated 03/2014
   d) Bathicillin, Teva UK Ltd. Last updated 25/02/2016
2. Martindale "The Complete Drug Reference" accessed via www.thomsonhc.com on 30/08/2013
3. American Hospital Formulary Service Drug Information 2011
4. ASHP 'Handbook on Injectable Drugs' 17th Edition pg 1150
5. British National Formulary No. 70, September 2015 pg 521 and 522
6. British National Formulary for Children 2015-2016 pg 342
   a) Evelina London Paediatric Formulary
7. MHRA guidance for healthcare professions on using and managing infusion systems
   a) Specimen High Risk Injectable Medicines List – November 2016
8. Development of the UK Vessel Health and Preservation (VHP) framework: a multi-organisational collaborative; 2016
9. a) Drug company name: Pfizer. Date contacted: October 2015
   b) Drug company name: Teva. Date contacted: September 2016
   c) Drug company name: Panpharma (PL holder Xellia). Date contacted: August 2016
10. Pfizer Material Safety Data Sheet 2007

Version MDJ3

Maximum bathicillin dose which can be infused in 1 hour, 80 minutes and 2 hours for patients of different weights:

**Weight (kg)**	**Length of infusion**		
	**1 hour**	**80 minutes**	**2 hours**
	Maximum bathicillin dose which can be given in infusion time		
40	120 mg	160 mg	240 mg
50	150 mg	200 mg	300 mg
60	180 mg	240 mg	360 mg
70	210 mg	280 mg	420 mg
80	240 mg	320 mg	480 mg
90	270 mg	360 mg	540 mg
100	300 mg	400 mg	600 mg
110	330 mg	440 mg	660 mg
120	360 mg	480 mg	720 mg
130	390 mg	520 mg	780 mg
140	420 mg	560 mg	840 mg

Unimycin

Date published: 21st September 2018

Intravenous - ADULT

Click on the icons for background information

Preparation and Administration Summary

Before treatment

Safety Alert

Double check the correct dose has been prescribed, because unimycin may be administered as a loading dose followed by a smaller maintenance dose (see 'Other comments' section below).

Commented [MJ16]: Pictogram to highlight safety alert, which some nurses missed/found with difficulty. Pictogram is a standard symbol for an alert.

Presentation of medicine [1]

Glass ampoules containing unimycin. Strength: 250 mg in 10 mL

Method of administration [1][5]

- **IV injection** or **short IV infusion** – for loading doses
- **Continuous IV infusion** – for maintenance doses

Preparation [1]

IV injection or **short IV infusion** – for loading doses:

- **Dilute dose to 100 mL with sodium chloride 0.9% or glucose 5%**
  1. Remove volume equivalent to the required dose from a 100 mL bag of sodium chloride 0.9% or glucose 5%
  2. Add required dose of unimycin injection to the bag

OR

Continuous IV infusion – for maintenance doses:

- **Dilute to a concentration of 1 mg in 1 mL with sodium chloride 0.9% or glucose 5%**
- For example, to make a 500 mg in 500 mL solution:
  1. Remove 20 mL from a 500 mL bag of sodium chloride 0.9% or glucose 5%
  2. Add 500 mg (20 mL) of unimycin injection to the bag

If patient is fluid restricted, you can give higher concentrations (or undiluted unimycin) by a central venous access device

Commented [MJ17]: Moved out of bullet point, as found with difficulty by four nurses.

Expiry time to write on label of continuous infusion [4]

24 hours

Administration [1][5]

- Flush with sodium chloride 0.9% or glucose 5%

IV injection or **short IV infusion** – for loading doses:

- Give slowly over at least 20 minutes, using an infusion pump
- Do **not** give faster than 25 mg per minute
- Give higher concentrations (or undiluted unimycin) via a central venous access device

OR

Continuous IV infusion – for maintenance doses:

- Infuse at prescribed rate using an infusion pump
- Give higher concentrations (or undiluted unimycin) via a central venous access device
- If necessary, calculate the infusion rate using the following equation:

$$\text{Unimycin infusion rate (mL/hour)} = \frac{\text{Prescribed rate (mg/hour)}}{\text{Infusion concentration (mg/mL)}}$$

- For example, for a prescribed rate of **35 mg/hour** using an infusion concentration of **1 mg in 1 mL**, the calculation is as follows:

Commented [MJ18]: Four users only found rate with difficulty. May be easier now summary removed, as this calculation will come sooner.

$$\text{Unimycin infusion rate} = \frac{\mathbf{35}\ \text{(mg/hour)}}{\mathbf{1}\ \text{(mg/mL)}} = 35\ \text{ml/hour}$$

- **Prescribers:** To calculate the infusion rate using the patient's body weight, see 'Example dose calculation' section below

Compatibility [1][4]

Compatible infusions (it is assumed that medicines meet close to the vascular access device):

- Esmolol
- Fluconazole, foscarnet
- Labetolol, linezolid
- Meropenem, micafungin
- Nicardipine
- Pancuronium, piperacillin-tazobactam, potassium chloride
- Tacrolimus, terbutaline
- Vecuronium

Incompatible drugs:

- Amiodarone
- Ciprofloxacin, clarithromycin, clindamycin
- Dobutamine
- Ondansetron
- Phenytoin sodium
- Salbutamol
- Vancomycin

Compatible infusion solutions:

- Glucose 10%, glucose 20%
- Hartmann's solution (compound sodium lactate)
- Ringers solution for injection
- Sodium chloride/glucose mixtures

Adverse effects and monitoring [1][5]

These adverse effects may occur during or shortly after IV administration:

- Hypotension, arrhythmias and convulsions – especially if given rapidly

- Hypersensitivity reactions
- Nausea and vomiting
- Dizziness, headache, central nervous system (CNS) stimulation and insomnia

Commented [MJ19]: Defined abbreviation (user suggestion).

If acute adverse effects occur, slow the rate or stop the infusion for 5-10 minutes

Monitor:

- ECG, heart rate and blood pressure
- Plasma-unimycin levels according to local policy
- Serum potassium levels if therapy is on-going

Extravasation

Likely to cause tissue damage due to extreme pH (see 'pH' section below)

Detailed Information

Other comments [(1)(2)(5)]

- A loading dose is not normally given to adults or children taking oral unimycin; if considered necessary, delay treatment until a serum unimycin level is available
- Store ampoules at room temperature (25ºC or below) and in original packaging to protect from light.
- Discard if the ampoule contents are discoloured
- Recommended dilutions are based on common practice in the UK

Example dose calculation

Continuous IV infusion – for maintenance doses:

- The initial maintenance infusion rate should be less than 500-700 micrograms/kg/hour (or 300 micrograms/kg/hour in older patients)
- Use ideal body weight in obese patients
- Calculate the infusion rate using the following equation:

$$\text{Unimycin infusion rate (mL/hour)} = \frac{\text{Dose (micrograms/kg/hour)} \times \text{Patient weight (kg)}}{1{,}000 \times \text{Infusion concentration (mg/mL)}}$$

- **Example:** For a **70 kg** patient on a maintenance dose of **500 micrograms/kg/hour** using a solution of **1 mg in 1 mL**, the calculation is as follows:

$$\text{Unimycin infusion rate} = \frac{\mathbf{500}\text{ (micrograms/kg/hour)} \times \mathbf{70}\text{ (kg)}}{1{,}000 \times \mathbf{1}\text{ (mg/mL)}} = 35\text{ mL/hour}$$

- **Click on this link for a table** showing unimycin infusion rates for selected patient weights (adults)
- The prescriber should adjust the rate and duration of the infusion according to plasma-unimycin levels and patient requirements

Latex content [(9a-b)]

These products are **not** made with natural rubber latex and have **not** been in contact with natural rubber latex during manufacture (Jul 15).

Sodium content [(1)]

Negligible (undiluted)

pH [(4)(9b)(12)]

- Undiluted: pH 8.6 to 10
- Diluted to 1 mg in 1 mL in sodium chloride 0.9% or glucose 5%: pH 9 to 9.2

Osmolarity / osmolality [(4)]

- Undiluted: 170 mOsmol/L
- 250 mg unimycin diluted in 100 mL sodium chloride 0.9% or glucose 5%: 290-320 mOsmol/L

Infusion pump performance [(7)]

- Infusion is 'Therapy Category' A due to narrow therapeutic margin
- The infusion pump used should have critical performance parameters described for 'therapy category' A

Product risk factors

Risk assessment of common preparations (as required by NPSA Patient Safety Alert 20):

Loading dose (adult): A risk assessment carried out on a dose of 300 mg unimycin in 100 mL sodium chloride 0.9% prepared in a clinical area identified the following risk factors: Therapeutic risk; Use of a concentrate; Use of part vial or more than one vial; Use of pump.

- Usual total risk factors: 4
- Overall risk rating: amber

Maintenance infusion (adult): A risk assessment carried out on concentration of unimycin 1 mg in 1 mL (500 mg in 500 mL sodium chloride 0.9%) prepared in a clinical area identified the following risk factors: Therapeutic risk; Use of a concentrate; Complex calculation; Use of part vial or more than one vial; Use of pump.

- Usual total risk factors: 5
- Overall risk rating: amber

Current suppliers

Concordia International - formerly AMCo
Supplies Mercury Pharma product
SPC for Unimycin hydrate 25mg/ml Solution for injection
PIL for Unimycin hydrate 25mg/ml Solution for injection

hameln pharmaceuticals limited
SPC for Unimycin Injection BP (Hameln)
PIL for Unimycin Injection BP (Hameln)

References

11. Summary of Product Characteristics
12. a) Mercury Pharma (Supplier Amidipharm Mercury); Unimycin hydrate 25mg/mL. Last revised 23/08/2012.
13. b) Hameln. Unimycin injection. Last revised 23/01/2015.
14. Martindale 'The Complete Drug Reference' accessed via MedicinesComplete on 27/05/2015
15. American Hospital Formulary Service 'Drug Information' accessed via MedicinesComplete on 27/05/2015
16. ASHP 'Handbook on Injectable Drugs' accessed via MedicinesComplete on 27/05/2015
17. British National Formulary Online accessed on 270/5/2015
18. British National Formulary for Children Online accessed on 27/05/2015
19. MHRA guidance for healthcare professions on using and managing infusion systems

20. a) Specimen High Risk Injectable Medicines List – November 2016
21. Development of the UK Vessel Health and Preservation (VHP) framework: a multi-organisational collaborative; 2016
22. a) Drug company name: Amdipharm Mercury. Date of contact: 07/07/2015
23. b) Drug company name: hameln Pharmaceuticals. Date of contact: 06/07/2015
24. Guy's and St. Thomas', King's College and University Lewisham Hospitals Paediatric Formulary 9th Edition accessed at www.guysandstthomas.nhs.uk/resources/publications/formulary/paediatric-formulary-9th-edition.pdf on 27/05/2015
25. NHS Lothian. Critical Care Guidelines. Unimycin. December 2012. Accessed on 27/05/2015 at www.nhslothian.scot.nhs.uk/Services/A-Z/CriticalCare/DrugsList/DrugsList/unimycin.pdf
26. Quality Assurance Department, Charing Cross Hospital July11

Version MDJ3

ADULT: Unimycin infusion rate table for selected weights

Maintenance infusion (using a solution diluted to 1 mg in 1 mL)

Weight Kg	Maintenance dose		
	500 micrograms/kg/hour	600 micrograms/kg/hour	700 micrograms/kg/hour
	Unimycin infusion rate (mL/hour) **using a 1 mg in 1 mL solution**		
40	20	24	28
50	25	30	35
60	30	36	42
70	35	42	49
80	40	48	56
90	45	54	63
100	50	60	70
110	55	66	77
120	60	72	84
130	65	78	91
140	70	84	98
